# Exploiting social influence to magnify population-level behaviour change in maternal and child health: study protocol for a randomised controlled trial of network targeting algorithms in rural Honduras

**DOI:** 10.1136/bmjopen-2016-012996

**Published:** 2017-03-10

**Authors:** Holly B Shakya, Derek Stafford, D Alex Hughes, Thomas Keegan, Rennie Negron, Jai Broome, Mark McKnight, Liza Nicoll, Jennifer Nelson, Emma Iriarte, Maria Ordonez, Edo Airoldi, James H Fowler, Nicholas A Christakis

**Affiliations:** 1 Division of Global Public Health, School of Medicine, Center on Gender Equity and Health, University of California San Diego, San Diego, California, USA; 2 CEO, EmpleApp; 3 Department of Sociology, Yale Institute for Network Science, Yale University, New Haven, Connecticut, USA; 4 School of Information, University of California Berkeley, Berkeley, California, USA; 5 Salud Mesoamerica Initiative, Inter-American Development, Washington, District of Columbia, USA; 6 Salud Mesoamerica Initiative, Tegucigalpa, Honduras; 7 Department of Statistics, Harvard University, Cambridge, Massachusetts, USA; 8 Division of Global Public Health, School of Medicine, University of California San Diego, San Diego, California, USA

**Keywords:** SOCIAL MEDICINE

## Abstract

**Introduction:**

Despite global progress on many measures of child health, rates of neonatal mortality remain high in the developing world. Evidence suggests that substantial improvements can be achieved with simple, low-cost interventions within family and community settings, particularly those designed to change knowledge and behaviour at the community level. Using social network analysis to identify structurally influential community members and then targeting them for intervention shows promise for the implementation of sustainable community-wide behaviour change.

**Methods and analysis:**

We will use a detailed understanding of social network structure and function to identify novel ways of targeting influential individuals to foster cascades of behavioural change at a population level. Our work will involve experimental and observational analyses. We will map face-to-face social networks of 30 000 people in 176 villages in Western Honduras, and then conduct a randomised controlled trial of a friendship-based network-targeting algorithm with a set of well-established care interventions. We will also test whether the proportion of the population targeted affects the degree to which the intervention spreads throughout the network. We will test scalable methods of network targeting that would not, in the future, require the actual mapping of social networks but would still offer the prospect of rapidly identifying influential targets for public health interventions.

**Ethics and dissemination:**

The Yale IRB and the Honduran Ministry of Health approved all data collection procedures (Protocol number 1506016012) and all participants will provide informed consent before enrolment. We will publish our findings in peer-reviewed journals as well as engage non-governmental organisations and other actors through venues for exchanging practical methods for behavioural health interventions, such as global health conferences. We will also develop a ‘toolkit’ for practitioners to use in network-based intervention efforts, including public release of our network mapping software.

**Trial registration number:**

NCT02694679; Pre-results.

**Strengths and limitations of this study**

- The sample includes a full population of individuals in 176 villages, 30,000 people.
- Measures capture comprehensive network data inclusive of a wide range of relationships.
- Intervention assesses the impact of network targeting on a variety of important reproductive, maternal, neonatal and child health health outcomes.
- A primary study outcome is a tool to allow interventionists to do network targeting with fewer resources.
- Limitations are that study results may depend on the geographical or public health setting.

## Background

### Neonatal mortality in Honduras

Despite global progress on many measures of child health, rates of neonatal mortality remain high in the developing world. Neonatal mortality now accounts for about 40–50% of under -5-years child deaths.^1^ ^2^ Intrapartum complications, prematurity and infections—including sepsis, pneumonia and meningitis—account for the majority of these deaths.^1^ ^3^ About 75% of neonatal deaths occur in the first week of life.^4^

Honduras has made considerable progress in its efforts to improve the health of it population^5^ but it still lags behind much of Mesoamerica. In Honduras in 2008, neonatal deaths accounted for 51% of all deaths of children under 5 years of age; 40% of these deaths were attributable to premature labour^6^ and another 40% were attributable to asphyxia and infection. Furthermore, 57% of all births occur in rural areas where perinatal care may be insufficient or unsafe.^7^ Although 79% of neonates start breast feeding within an hour of birth, only 30% are exclusively breast fed for the first 6 months of life.^5^

Although many deaths can be prevented through provision of clinical care services, emerging evidence suggests that a substantial reduction in poor reproductive, maternal, neonatal and child health (RMNCH) outcomes can also be achieved with simple, low-cost interventions within family and community settings.^8–10^ The challenge is how to implement interventions in low-resource environments where health systems are often weak, and where intervention delivery is often dependent on short-term funding cycles.^11^ Another equally fundamental question is how these interventions might be delivered so that communities actually adopt the behaviours being promoted, and how to ensure that those behaviour changes are sustained. To promote improvements in key RMNCH behaviours, change is needed in the provision of service and also in community-level demand for service and practices.

**Figure 1:**
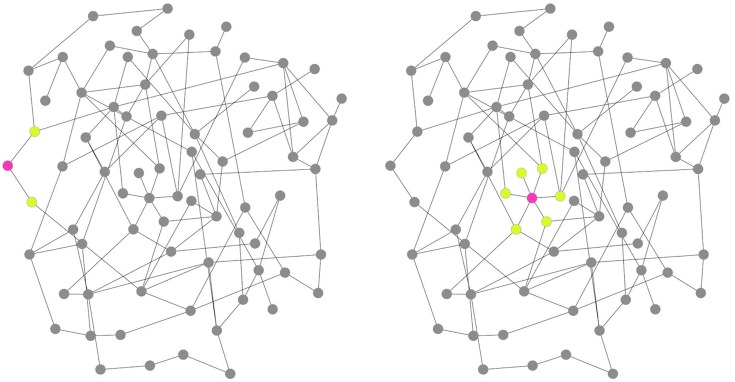
Variation in structural position in a network. Different individuals are typically able to exert variable amounts of social influence depending, in part, on both their number of connections and their location within the larger social network. The person in the left panel (red node) has two network ties (degree=2) and occupies a peripheral position in the network. In contrast, the person in the right panel has six connections (degree=6) and holds a central position in the network. The extent of potential spill-over effects a person may induce is generally likely to be higher for the node on the right than for the node on the left.

Behaviours related to RMNCH care are often socially reinforced, and can therefore be difficult to change, particularly in traditional cultural settings.^8^ In these contexts, the study of norms, influence and social position are important to understand the functioning of interventions attempting to improve RMNCH outcomes. Social network analysis can be used to understand these dynamics. In particular, two distinct but interacting social network mechanisms can affect health decisions: contagion and connection.^12^ Contagion refers to the spread of a behaviour from individual to individual. Connection refers to the standing of the individual within the wider social structure (see figure 1). While exposure to a new idea or behaviour through social contacts can influence an individual to change one's behaviour,^13^ an individual's overall position within the larger network can also impact the possibility of behaviour change.^14^ For example, people who are on the periphery of a network may have less access to important resources, simply by virtue of where they are positioned within the network, while people at the centre of the network may be less willing to change behaviour because their actions are under greater scrutiny.^15^ ^16^ In other words, social position can often promote or hinder exposure to new ideas, while the social cost inherent in adopting a new behaviour can also differ according to one's position in the network. Those most socially central, for example, may be those who are expected to uphold strongly established norms.^12^

Social networks are therefore a highly relevant substrate for the impact of social norms.^12^ ^17^ ^18^ A social-norms perspective on behaviour change considers the choices of individuals to be significantly affected by the behaviours and/or opinions of those in their salient reference groups*,* or those to whom an individual turns for expectations regarding behaviour.^19^ ^20^ If social norms are the driving force behind behavioural decisions, looking at the structure of a community's social networks might help us to better understand why individually focused behavioural interventions may be ineffective. Since the transmission of norm-changing behaviour (such as those promoted by many community interventions) often requires multiple reinforced exposures, it can be initially inhibited by highly connected networks and yet also ultimately requires the reinforcement of such networks to successfully occur.^21^ ^22^ The effect of injunctive norms, or what people believe is approved of within their reference group,^23^ may be more powerful in highly interconnected communities. The promotion of ‘acceptable’ behaviours and sanctioning of ‘unacceptable’ behaviours may be stronger.^18^ ^24–26^ Yet, this dynamic also works inversely, such that when a critical mass of a highly interconnected group has adopted a behaviour, the probability that any individual in that group will adopt also increases.^21^ ^27–29^

Owing to the powerful impact of social networks on human behaviour, social network targeting (where structurally influential individuals are selected for the receipt of interventions) shows great promise in the implementation of sustainable community behavioural change. Recent work by our group in a different part of Honduras has demonstrated that interventions targeting friends of randomly selected individuals might be more effective than interventions targeting those individuals themselves.^30^ A school-level intervention on bullying achieved the greatest reduction in student conflicts through the targeting of ‘social referents’, or those within the top 10% of individuals according to how many friendship nominations they received.^31^ Smoking and alcohol cessation programmes that exploit peer influences that modify the social network of the target have been shown to be more successful than those that do not.^32–38^

In sum, social network targeting represents a paradigm shift in how we currently implement interventions in global health settings. Many behaviour-change interventions currently seek to target all members of a population; however, face-to-face counselling for behaviour change takes time and resources. Ideally, successful social network interventions methodologies could mean that intervening in smaller segments of the populations will have the same effect as targeting 100% of the community, saving considerable time and money.

### This study

Our objective is to conduct a randomised, controlled trial (RCT) of novel social network targeting techniques in order to explore how social network dynamics affect the uptake, diffusion, and group-level normative reinforcement of key RMNCH behaviours and attitudes in rural Honduras. Health behaviours will be promoted through a community-level household-based intervention that will be implemented by the Inter-American Development Bank (IDB) through the Salud Mesoamerica Initiative (SMI), with whom we are partnered for this project. We will use theoretically derived algorithms to choose a subset of structurally influential individuals from within the population to receive an intervention. Throughout this protocol the term ‘treatment’ refers only to the algorithms used to choose individuals, while the term ‘intervention’ refers only to the programme designed to promote positive RMNCH behaviours and attitudes. Our ‘treatment’ here is not the intervention we are using, but rather the algorithms used to choose a subset of structurally influential individuals from within the population receiving the intervention. We will assess whether differential network targeting results in differential uptake (ie, differential practice) of the RMNCH behaviours and attitudes promoted in the intervention.

**Figure 2:**
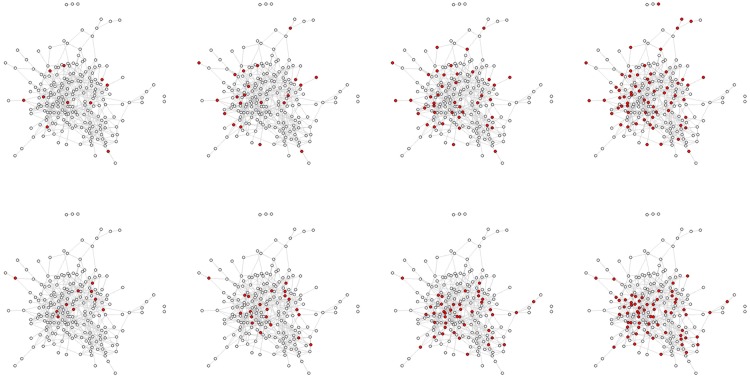
This figure displays a network map of a real village with 206 inhabitants in Honduras. The top row displays individuals selected at random (in red); the bottom row displays individuals selected by the ‘friendship nomination’ technique (they are a single randomly chosen friend of the randomly chosen individuals) (also in red). The columns display 5, 10, 20 and 30% targeting from left to right. It is apparent that (1) at the same percentage, friends of randomly chosen individuals are more central in the network and have higher degree than the random individuals, and (2) that, as the sampling fraction rises, the difference between the random nodes and the friends nodes declines (as is expected given network theory).

We will randomise 176 villages using an 8×2 factorial design in which we will (1) vary the proportion of people targeted per village in 8 distinct groups and (2) compare random targeting to targeting friends of randomly selected people (see figure 2). Since we are intervening at the household level, our random targeting versus friend targeting randomisation will also be carried out at the household level. (Details regarding our randomisation strategies are outlined in Analytic Aim 2 and 3 and in online supplementary appendix 1). We will then measure changes in behavioural and attitudinal outcomes for individuals and communities including both those who got the intervention and those who did not. Our results will allow us to assess how the difference in adoption of interventions, at both the individual and community levels, varies across the various arms of the trial.

10.1136/bmjopen-2016-012996.supp1 supplementary appendix

Specifically, we will address:

1. Can the structure of social networks provide clues regarding subpopulations at higher risk of experiencing RMNCH morbidity and mortality or at higher risk of being unresponsive to behaviour-change interventions?
2. Based on network parameters, can we exploit mathematical algorithms to identify a well-positioned set of people who exert the maximum influence on adoption of improved RMNCH care practices in the larger population, and thus have a multiplicative effect on coverage?
3. Can we, in parallel, identify sets of people who are responsive to such influence (ie, influenceable and not just influential, people)?
4. Is there a threshold effect such that when a certain proportion of the population has changed their norms around key behaviours, the rest of the population is likely to follow?

**Figure 3:**
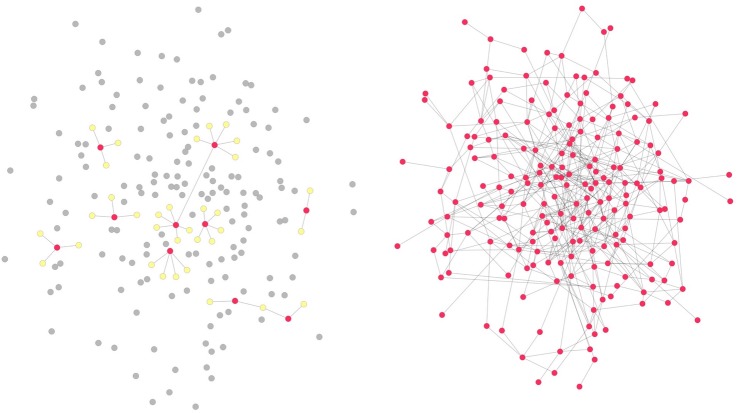
Illustration of network sampling. The left panel shows a network obtained through egocentric sampling. An egocentric sample consists of a set of sampled ‘egos’ shown as red nodes (the individuals whose characteristics are being studied) and a set of ‘alters’ shown as yellow nodes (the individuals who were nominated by the egos). Only ego-alter ties and some (typically very small number, if any) ego–ego ties are observed in an egocentric study, leaving all alter–alter ties outside the sample (excluded nodes shown in grey). In contrast, a sociocentric study design, such as the one proposed here and shown on the right, enables observing all existing ties among the sampled set of nodes.

A key feature of this research is that the network data collection we are proposing is sociocentric rather than egocentric. As illustrated in figure 3, egocentric data, while it involves social network information, is collected from a sample of individuals within a given population.^39^ ^40^ In contrast, sociocentric network data creates an image of a collective whole, with comprehensive data gathered on ties between all of the people within a specified population.^39^ ^40^ Whereas egocentric data may help to improve the representativeness of a sample for a large population, sociocentric data allows measurement of larger network structures (like network communities) and individual-level network measures based on them.

## Methods/study design

### Preliminary work

As preparation for this project, we completed geographical mapping of over 200 villages in the study region, allowing us to gain a more precise understanding of the study population and field conditions, including terrain, rainfall and distances to health facilities, to inform planning and implementation. We developed an extensive survey instrument to capture the various outcomes that will be addressed through the household-level interventions that will be implemented by IDB/SMI, including use of folic acid, prenatal care utilisation, birth plan preparation, immediate breast feeding after birth, exclusive breast feeding for infants up to 6 months old, proper thermal and cord care for newborns, proper treatment of diarrhoea in children and paternal involvement in childcare. Our survey instrumentation is primarily composed of validated scales used widely to measure items related to RMNCH outcomes. We conducted an extensive review of the RMNCH literature and consulted global RMNCH experts for their advice on the inclusion of appropriate items in the survey. We also did extensive formative research, including detailed qualitative individual interviews and focus groups, and cognitive interviewing to assess our survey's cultural relevance and consider regional language variations specific to the study area. In addition, we conducted three rounds of pilot data collection involving network mapping and sociobehavioural interviews to test the network questions: our newly designed social networks data collection software, (named ‘Trellis’; see online supplementary appendix 2): and the behavioural health outcomes instrumentation (for more details on our pilot work and field operations please see online supplementary appendix 1).

10.1136/bmjopen-2016-012996.supp2 supplementary appendix

### Study population

Our study is being conducted in the department of Copan, Honduras, in an area of over 200 square miles of rugged mountainous terrain with an estimated total population of 92 000 people. In order to power the 8×2 design (for details please see Aim 2), we have selected 176 villages from the 238 small towns and villages located in this area. Factors like population size, accessibility and safety were considered when selecting the final list of villages. Owing to high adolescent birth rates in this population, all individuals over the age of 12 who live or work in the study villages are eligible to enrol (see online supplementary appendix 1 for more detailed information). Individuals who are cognitively impaired and unable to provide consent are excluded. We have already conducted a photographic census in the 176 study villages. Recruitment rates are high: census data show ∼32 500 eligible individuals in these villages, of whom at least 93% (N=30 460) have agreed to enrol in our study. Using the Trellis software (please see online supplementary appendix 2), we obtained demographic data, photographs and Global Positioning System coordinates of all participants who enrolled. The average age for participants is 33, and most are married or in a civil union (59%), and slightly over half (54%) are women. The total number of respondents surveyed per village range between 55 and 620 individuals and the average household size is 2.8. We have not yet begun the intervention.

### Network data collection

As the photographic census is complete, we will next use bespoke software we have prepared and made publicly available (http://humannaturelab.net/resources/software/trellis/), named Trellis (see online supplementary appendix 2), to undertake the main survey, which includes a battery of ‘name generator’ questions to capture social relationships. The name generators will focus on several different types of affiliations including friendship, professional contacts, kinship, and contacts relevant to health behaviour. In this study, the boundaries of each network will be the village, so that individuals may nominate any individual from within their own village as a social contact. The photographs taken in the preliminary census will be used to validate the social contacts named by the respondents. Online supplementary appendix 1 provides additional technical details about social network measurement.

### Measurements

1. Baseline social networks: The name generator questions to collect sociocentric data will be included in the baseline survey. From these measures, we will calculate community-level and individual-level measures of network connectivity including various measures of centrality (please see online supplementary appendix for more details). (Wave 1 2016)
2. Baseline RMNCH care behaviour and norms for all individuals in the villages. Specific maternal, neonatal and child health behaviour questions will be asked only of individuals who have already had a child. Norms and attitudes questions will be asked of everyone and will include attitudes towards RMNCH behaviours as well as the role of fathers in prenatal and neonatal health. (Wave 1 2016)
3. Concurrent norms and behaviour surveys: 1 year into the intervention, we will administer surveys to track changes in norms and behaviours as well as possible sources of intervention spread. For this survey, we will also monitor the implementation of the intervention by asking questions specific to receiving intervention activities. (Wave 2 2017)
4. Final outcome survey: a second round of social networks data collection, RMNCH care behaviour and norms questions for all individuals in the villages. Using this second round of social network data, we will recalculate network connectivity at the individual and community levels. (Wave 3 2018)
5. Contextual surveys: We will gather data on contextual factors within each village, such as the size, distance and characteristics of the nearest clinic, other intervention activities that may be taking place through local organisations or non-governmental organisations (NGOs), important geographical features, facilities and infrastructure available to residents, etc.
6. Health outcomes: For a subset of families, our surveyors will specifically measure and record temperature and respiratory rates for children under 5 years.
7. Clinic records: We will collect clinical data from birth records from the regional maternity clinic for women who give birth at this facility. We will also collect postpartum health data from the local health centres. Postpartum data is recorded by family health teams conducting home visits with postpartum women in the villages within 7 days of delivery, regardless of birth location. Postpartum health records contain information on newborn weight, respiratory rate, temperature, head circumference and signs of redness, pus or swelling in the umbilical cord,^41^ as well as body temperature, blood pressure readings and presence of danger signs for the mothers.

### Behavioural health intervention

The implementing partner in this project, IDB through SMI, is responsible for designing and implementing an integral intervention to promote RMNCH behaviour change in Honduras. To work within the constraints of this study, the intervention had to meet specific requirements including: (1) alignment with priorities of the Ministry of Health (MOH) of Honduras, the Bill and Melinda Gates Foundation, and the needs of the population; (2) contain new messages for the targeted population to allow for detection of changes in knowledge, attitudes and practices; (3) include tracers or identifiers which could be detected during follow-up surveys; (4) not use mass-media communication techniques, including radio spots, flyers, posters, etc, as these would contaminate the network effects of the study; (5) have a strong monitoring component; and (6) have a demonstrated effectiveness in similar settings in order to test the spread of behavior from person to person. The intervention would also have to adopt the targeting strategy, focusing on network position, as opposed to targeting households with primary audiences for the behaviour change of interest. For example, the targeting algorithm could hypothetically identify a household with two grandfathers and therefore select it to receive the intervention. This household would usually not be selected for an intervention on prenatal care or neonatal practices, given that there are no pregnant women living there but we would include it.

Given these requirements, an educational package, using the Timed and Targeted Counselling (ttC) methodology^42^ complemented with alternative methods of face-to-face communication including songs, rhymes and riddles, was designed with World Vision and Child Fund Honduras. The social and behaviour change communication strategy for the intervention was designed using the ‘P-Process’, a tool developed by Johns Hopkins Center for Communication Programmes and used for more than 30 years for planning strategic, evidence-based health-communication programmes.^43^ This intervention will be delivered by trained community health workers for 24 months on a monthly basis to the households selected for the study. Timed and Targeted Counselling (ttC), has been implemented in 20 countries worldwide by World Vision.^42^ It is targeted in time (when it is needed), in space (by visiting in the home), and in individualised approaches (messages and strategies to remove barriers depending on the circumstances of a specific family). This methodology uses narrative and negotiation in a 1–2 hour visit with families to discuss positive and negative scenarios and create a list of agreements with families to try out new practices. It should be noted that ttC is normally implemented in households with pregnant women and/or children under 2 years, and counselling is provided to all family members based on stage of the pregnancy or age of the child.

This methodology was adjusted to include messages for topics of interest to the study based on findings from formative research conducted for intervention design (please see online supplementary appendix 3 for details) and evidence-based, cost-effective practices^2^ ^44^ related to study outcomes.

10.1136/bmjopen-2016-012996.supp3 supplementary appendix

Study outcomes include: (1) use of folic acid in women of reproductive age to prevent birth defects; (2) receiving prenatal care in the first trimester; (3) having a birth preparation plan for seeking timely prenatal care, institutional birth, postpartum care and emergencies; (4) exclusive breast feeding for infants under 6 months; (5) immediate breast feeding after birth; (6) proper thermal and cord care for newborn infants; (7) proper treatment of diarrhoea in children, including the use of zinc (which is a new component of the SMI programme, here); (8) paternal involvement in childcare, particularly for newborns; (9) use of modern family-planning methods; and (10) delaying pregnancy until 18 years of age.

While only very specific households will be targeted according to our randomisation methods, participants from target households will not be discouraged from inviting others, and a careful record of attendees will be kept. CHWs have a team of supervisors for intervention quality control and to ensure that CHWs are visiting the correct houses. The IDB/SMI team has incorporated the use of mHealth tools to aid in intervention delivery (eg, all stories are available in an animated video format), data quality and timeliness. The IDB is also currently working on supply-side interventions with the Government of Honduras through SMI and other complementary programmes, to ensure that community members seeking services receive quality care.

As part of the intervention protocol, we will keep careful track of which CHWs have visited which households in order to allow us to evaluate possible provider effects relevant to the study outcomes. For more details on intervention implementation please see online supplementary appendix 3

**Figure 4:**
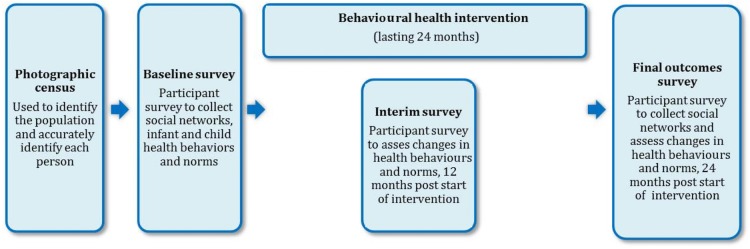
The sequence of events in this study.

Figure 4 illustrates the sequence of events in our project.

### Analytic aims

We have several analytic aims, as this project is analytically very complex (for details on statistical methods specific to each aim please see online supplementary appendix 1). The extensive amount of network data will allow us to undertake an unprecedented level of analysis regarding the relationship between community-level social dynamics and the uptake, diffusion and maintenance of intervention behaviours, and norms. These network data will be used to examine how attitudes and behaviours spread across network ties.

### Analytic Aim 1: evaluate the extent to which behaviour change regarding RMNCH care spreads

We will use the results of the randomised controlled trial (RCT) to ascertain the extent to which beneficial or harmful RMNCH care attitudes and behaviour change in one person can potentially modify the attitudes and behaviour in other people to whom they are connected. With the longitudinal data that we will collect in this study, we will be able to track the change in attitudes and behaviour among individuals over time. By examining the correlation between the behaviour of an individual and the behaviour of those to whom she is linked, we will be able to determine to what degree the intervention effects spread beyond those who were directly exposed to those who were not exposed (a ‘cascade’ or ‘spillover’ effect).

Here, our work benefits from experimentally exposing individuals to the intervention. This randomisation will allow us to assess causal relationships between connected individuals by measuring how participants' outcomes are affected by their social contacts' (randomly assigned) exposure. Across villages, we will assess how varying the overall treatment rate causes both treated and untreated individuals to alter their behaviour.

The magnitude, and possibly even the direction, of network effects should vary according to the social distance between the actors. In general, our previous research has demonstrated weaker effects with increasing social distance, so that, for example, a behaviour change (eg, performing hygienic cord care) in a social contact would have progressively weaker effects in terms of motivating behaviour change as one moves along, say, the continuum from sibling to friend to neighbour. Among socially connected individuals, we will be able to distinguish among mutual relationships (when both nominate each other) and ‘one-way’ relationships (when nominations are not reciprocated). We expect that social contagion will be more likely between mutual relationships compared to one-way relationships.

### Analytic Aim 2: test for social effects in community adoption rates

**Figure 5:**
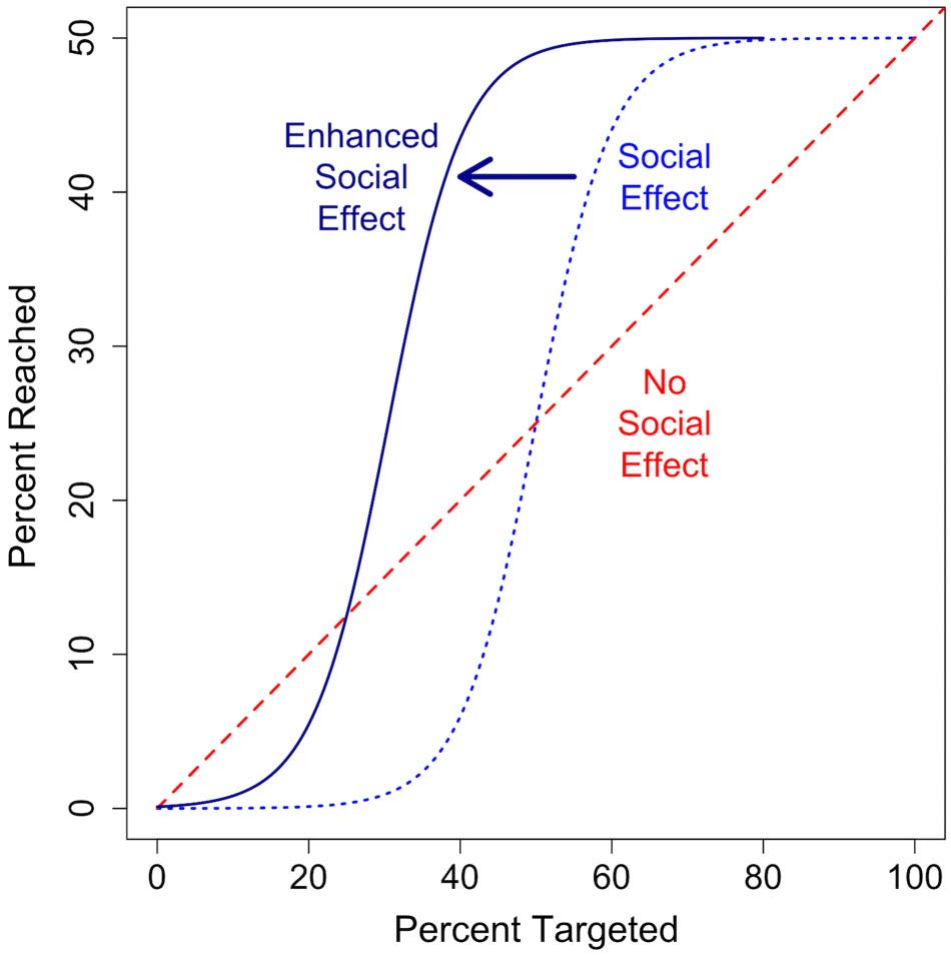
This figure illustrates possible results. The X-axis denotes the fraction of a village targeted for an intervention, and the Y-axis denotes the fraction that ultimately adopts the intervention. The red dashed line denotes the results for no social effect. Each person targeted has an equal chance of adopting regardless of the number of others treated. The blue dotted line denotes the results for a social effect. If we target people at random for an intervention, many of them may be reluctant to change their behaviour when few others have, so that the intervention is less effective per-person until a critical threshold is reached. At that point, adoption is more likely because of social reinforcement and the per-person effect of each targeted individual grows exponentially. Eventually, so many people have adopted that there is no willing person left to adopt and the per-person effect decreases once again, approaching 0. Understanding this dynamic is important, since even high targeting percentages that fall below the critical threshold might yield low adoption. Similarly, if there is a social reinforcement effect, it may not be necessary to target everyone. In the example above, targeting 60% of the individuals capture nearly 100% of the total possible intervention benefit. Finally, the dark blue solid line denotes results when we enhance the social effect through friendship targeting. If targeted people are well-connected, there will be greater exposure to the intervention through diffusion, shifting the whole S-shaped curve to the left. It takes fewer people to reach the critical threshold, and is possible to reach saturation with a smaller percentage targeted. Note that the Y axis denotes 0–50% and the X axis includes the full 0–100% as we assume there will be an upper limit on adoption associated with any particular intervention (here, we arbitrarily chose 50%).

Most interventions are focused on individuals. Researchers identify a group of individuals to enrol in a project, randomly assign some to the intervention and some to control, and then test the efficacy of the intervention on treatment individuals versus control. Individuals may be chosen from one defined community or from many. The impact of the intervention on the community as a whole is usually not measured or taken into consideration. However, we know that, for behaviours with a social component, the individual is not thinking or acting in a vacuum. As more and more individuals in a community are exposed to new behaviours and new ideas, it is increasingly likely that any given individual in that community will adopt those behaviours and ideas (see figure 5). There is a threshold to this effect, however, beyond which exposing additional individuals will not increase overall adoption. Analytic aim 2 is to learn where this critical threshold lies in order to capitalise on the social effects that create it. It may be possible to achieve near-maximum adoption with substantially less effort than is currently being exerted by interventions.

We plan to test for this threshold by using an 8×2 RCT factorial design. In the first dimension of this design, we will assign villages to one of eight dosage treatments (0%, 5%, 10%, 20%, 30% 50%, 75%, 100%) indicating the percentage of households in each village that will be randomly chosen to receive the intervention package (for details on randomisation methods see online supplementary appendix 1). We will use these randomly assigned treatment percentages and observed rates of health behaviour adoption (in the entire communities) to test for this threshold. What is the minimum proportion of the population to target in order to achieve maximum levels of adoption at the community level? Since we have several outcomes on which we are intervening, we will be able to test to what degree this threshold differs (or does not differ) across behaviours, and how this may differ according to the behaviour-change mechanism we observe (with due attention to multiple comparisons).

The two mechanisms by which we believe social networks affect intervention adoption are through the spread of information and the spread of norms.^21^ On the one hand, the spread of information can be understood as being a process of social learning^45^ where people mostly observe others adopting the behaviour and assess the benefits that the early adopters may be enjoying. On the other hand, the spread of norms is more likely to occur as the result of direct social influence.^45^ If the innovation is in direct opposition to an ingrained norm, community members may sanction others for adopting the new behaviour. The proportion of the population that must be exposed in order to overcome that resistance might be higher than it is for a diffusion of technology or knowledge. Once a critical mass adopts, however, and a new norm has taken hold, then we would expect that social influence would switch and work strongly in support of the innovation. In figure 5, at the higher levels of adoption, there is strong upward influence pushing the community in support of the behaviour of interest.

Diffusion and norms processes will both yield the S-shaped curve shown in figure 5, though potentially occurring at differing rates. Elucidating the difference between the two is crucial; however, the intervention strategies necessary to elicit successful change will differ between the two. To further distinguish these two mechanisms, we plan to generate questions in our survey that will help us to differentiate whether a health behaviour showing a social effect is influenced more by information transmission or through social influence and social sanctions. We will also analyse social structural features that affect this (see online supplementary appendix 1).

### Analytic Aim 3: test the impact of the ‘nomination’ network targeting method

If people who are initially selected to receive the intervention are socially well connected, it follows that, because their local social networks are larger (and for other topological reasons as well), the number of people who will be exposed to the intervention at an early stage, through spill over, will also be larger. Moreover, if we identify these central people through the most topically salient relationships, they might also be people with high credibility around our behaviours of interest. In other words, we anticipate that these individuals will not only have more influence in the community due to their greater level of access to others, but that the probability that any individual within their network adopts might be greater due to their higher level of general credibility.^46^ Theoretical network research supports both these hypotheses,^47^ ^48^ but there is little empirical evidence to test these claims.^30^

Hence, a unique dimension of this study is that we will vary how initial targets are selected. In our 8×2 factorial design, the ‘8 conditions’ differ according to proportion of the community targeted (see Aim 2). The ‘two conditions’ differ according to how households within that community are chosen. In one condition, individual households are selected at random; in the other condition, households are selected using the ‘friendship nomination’ procedure that will identify individuals who are more connected in the village.^49^ ^50^ In the friendship nomination condition, we will randomly select individuals and then, rather than intervene on those randomly selected individuals and their households, we will intervene on the household of one of their nominated social contacts. Past work suggests that the adoption curve under nomination targeting is shifted to the left relative to random targeting because friends are more central in the network and therefore spread information and norms more quickly in the early part of the intervention.^30^ This shifts the whole adoption curve to the left, as shown in figure 5 (the ‘Enhanced Social Effect’ shown by the solid dark blue line), and, as a result, the total percentage of individuals that need treatment to achieve nearly-maximum adoption also shifts to the left. The consequence of this left-shift is that intervention adoption increases for the same number of initial targets. This increased efficiency through the social effect is a central and overarching study objective.

### Analytic Aim 4: evaluate the impact of network structural characteristics on behaviour change

We will ascertain how network structure (at the individual level and at the village level) moderates the impact of the intervention. Do people in certain parts of the network, as measured by network parameters—including centrality, transitivity and clique membership (see online supplementary appendix 1 for more details on network structural measures)—respond differentially to intervention? How does a person's location in a network, above and beyond their personal attributes, affect their response? We will also use this information to identify as-of-yet undiscovered network structural-risk factors for RMNCH morbidity and mortality. This may allow us to make important recommendations for targeting specific parts of the network in future interventions.

Since we are collecting sociocentric data (see figure 2), we will have the ability to measure a wide range of structural features to determine whether they moderate the effect of the RCT intervention. For example, is the randomly assigned intervention more effective for people in the centre of the network than it is for those in the periphery? Is the intervention more effective for those in denser parts of the network with more cross-cutting ties or for those in sparser, less-connected parts of the network? With respect to community structure, do large or small clusters in the network experience better outcomes?

Results of these analyses will include: (1) network measures for all individuals within the network and higher-order features like clusters and communities for the overall network itself; (2) information regarding the relationship between network measures and outcomes of interest, including any possibly moderating effects; and (3) recommendations for using network measures, including network position and network subgroups, for future interventions.

### Analytic Aim 5: ascertain whether partial collection of network data can identify the most influential or the most influenceable people in the network

Ultimately, a key goal of this RCT is to develop means by which network approaches can be used to design, implement, and monitor health interventions most effectively. We intend to develop means by which network strategies can be incorporated into health behaviour programmes without having to map the entire network. While understanding the interaction between social relationships and intervention uptake is undoubtedly of great theoretical interest, ultimately this work is about the application of network tools to real-world projects. In real-world scenarios, the mapping of entire networks is likely to be expensive and time-consuming. A successful demonstration of the friendship nomination technique for identifying influential individuals within networks (which can be implemented without mapping the whole group) will help address this.

To further address the issue of scalability, one of our analytic aims is to use the full network data in this project to develop means by which interventionists can collect partial network data to achieve the same outcome, but at much lower cost. Recent research has suggested that, properly implemented, random network sampling can be used as a proxy for complete network information.^51^ For example, in our previous work, we showed that it was possible to predict an outbreak of H1N1 influenza in a population 6 weeks in advance, even without measuring the full social network.^49^ While health behaviour interventions probably spread differently than infectious diseases, the rationale behind using a network-based approach to disseminate an intervention, particularly one relying on central actors, has been proposed in a wide range of fields. Indeed, network-based approaches to health behaviour interventions using ‘opinion leaders’ are increasingly common in the developed world.^47^ ^52^ ^53^

### Analytic Aim 6: assess the effect of the intervention on the village social networks

We will ascertain whether and how the interventions may actually modify the actual network structures of the villages. Does the introduction of a health intervention to a village change social interactions? And what are the possible effects of these changed structures on the health of communities or the population?

While programme monitoring and evaluation typically focus on the intended objectives of a given programme, interventions can have unanticipated outcomes which are rarely measured or published in the peer-reviewed literature.^54–57^ Nevertheless, it is critical that interventionists consider unintended outcomes, whether positive or negative, in order to integrate these outcomes into cost-benefit analyses for programme planning.^58^

One of the most potentially profound, but largely overlooked, peripheral outcomes of health promotion interventions is a fundamental change in social structure as a result of the intervention activities. Previous network research has demonstrated that social marginalisation can occur among people who violate the norms of their proximal networks.^59^ ^60^ As norms change due to the effects of well-intentioned interventions, the social landscape of the population in question can change as well. For example, an intervention designed to increase academic performance among air force academy students failed because the intervention unintentionally created segregated clusters of very high and very low ability students, which reinforced the academic challenges of those same very low ability students that the intervention was designed to assist.^61^ Our past research (in the USA) has shown that social isolation of smokers increases the likelihood that they will cluster together, which further reinforces their smoking behaviour.^62^

It is possible that those embracing the novel behaviours in our RCT may form new connections based on their mutual interest in the intervention and, as a result, help create clusters of ‘adopters’ within the network. When adopters create clusters, non-adopters may be left to form their own clusters. Examining to what degree this occurs can inform future interventions and ameliorate any possible unintended health disparities that might result from an otherwise successful intervention. The intervention may also strengthen the social health of the community, yielding unintended but beneficial effects on a wide range of health outcomes other than RMNCH-related outcomes.

To the best of our knowledge, this will be the first large-scale evaluation of how an RCT in a developing world setting possibly modifies social interactions and what impact this modification has on health. We will assess changes in egocentric and sociocentric network structures from wave 1 to wave 3 (when the network is mapped for the second time, roughly 2 years later), and compare those changes across the different arms of the trial, examining whether there is a significant difference in the evolution of the networks for villages exposed to the intervention versus those that are not exposed. Specifically we will be looking for: (1) the emergence of clusters of intervention adopters, (2) the emergence of clusters of intervention non-adopters and (3) large-scale, and ego-centric, changes in the overall network (such as increased density).

## Discussion

Our study is unusual in that it will allow us to both (1) understand the way in which social network dynamics affect the uptake of a large scale RMNCH intervention, and (2) evaluate how network-based targeting can maximise the overall impact of the same intervention. We will use a detailed understanding of social network structure and function to identify novel ways of targeting influential individuals so as to foster behavioural cascades and population-level behaviour change.

We will achieve this objective by conducting a randomised controlled trial of network targeting algorithms, to be deployed in a sample of 176 villages in Honduras, with a 2-year package of monthly RMNCH care interventions. Our work will involve both experimental as well as observational analyses, and it will be one of the largest efforts to map face-to-face networks of which we are aware (involving over 30 000 people). We will test a scalable method of network targeting that would not, in the future, require the actual mapping of social networks, but that would still offer the prospect of rapidly identifying influential targets for public health interventions. If successful, we will have developed procedures that will allow us, and others, to accelerate the change of attitudes and behaviours in entire populations much more efficiently and comprehensively.

### Limitations

Our study has limitations. Our sample is limited to rural Honduras and so, while many network characteristics tend to be similar across different social and cultural contexts,^63^ some of the norms surrounding RMNCH care might be special. This may limit the application of some of our results within other contexts. Also, while we will use objective measures whenever possible, many of the outcomes we will be assessing will be measured using self-report. Finally, while between-village ties would strengthen our understanding of network dynamics within this population, the extensive resources involved in that level of data collection preclude our ability to collect that data (though we forecast such ties to be less relevant and less numerous).

Our intervention itself also has limitations. In the ideal scenario, we would use an evidence-based intervention for community-based neonatal health tested in rural Honduras implemented to ‘guarantee’ behaviour change among the initially targeted individuals, the ripples of which would be studied in the RTC. However, given the constraints of the study, this ‘ideal intervention’ does not exist. For example, in the ideal behavior change communication intervention, the person or group whose behaviour change is sought receives the intervention messages as many times as possible. In the typical community-based intervention, in addition to face-to-face counselling, community members would be exposed to radio messages, banners, flyers, mass text messages and other media-based communication methods to reinforce messaging. Given that the study relies on information passing through the social network, mass media communication are being excluded. The intervention team has been creative in adding tools to the intervention, and has been mindful of including a variety of behavioural changes along the continuum of pregnancy, childbirth, postnatal care and child health in order to maximise the possibility that one or more intervention components are adopted. Intervention targeting is also affected. Normally in an intervention targeted at changing behaviours in maternal and neonatal health, households would be selected depending on where pregnant women live. We could not take these criteria into consideration, given that the targeting mechanism is based on position in the network, rather than whether or not a woman in the household is pregnant.

Finally, not all aspects of the desired behaviour change rely on adequate knowledge, attitudes, and practices at the household level. A clear example is how the conditions in health centres and hospitals affect behaviours of the population. Although messaging is provided to families regarding the importance of male involvement in birth, if the hospital or health centres do not have adequate infrastructure to have private birthing rooms (the norm in Central America), men cannot be in the room during the birth if another women is also in labour. Although SMI works closely with the MOH to improve supply-side conditions, some aspects are out of the scope of the programme. The study team is aware of these limitations and is documenting them to have a clear picture of these external factors which also impact the success of the community-based behaviour change intervention.

## Conclusion

This study is unique in its scale of network data collected in a developing world setting, the integration of complete network data with the results of a large RCT, and, most importantly perhaps, the testing of network algorithms as a method of boosting uptake of the intervention. With billions of dollars spent each year in attempts to achieve behaviour change in at-risk populations, we are still unsure of the best strategies for implementing these interventions in ways that maximise sustained change. This study will be an important contribution towards that goal, with results that can be applied in disparate global contexts.

We believe that the knowledge gained will have substantial practical application. Global health practitioners are beginning to understand that ignoring social reinforcements and expectations in relation to the outcomes of interest can herald less -than- optimal results. It is not clear however, how to apply that understanding to the practicalities of intervention work. By looking at behavioural and attitudinal outcomes of our intervention, we have the opportunity to take a real-world, on-the-ground intervention and analyse it in conjunction with gold-standard, complete social network data. By exploiting network insights, we will be able to develop strategies for interventionists to implement in order to shift the norms of the communities towards acceptance and uptake of the desired behaviours.

### Dissemination

The primary applied aim of this project is to develop methods by which interventionists in global health settings can integrate a network approach in order to maximise the effectiveness of their programmes. The analytic aims we have outlined above are essential for achieving this goal. Analysis and publication in peer-reviewed journals alone, however, will not make this body of knowledge accessible to those for whom it is intended, namely, global health practitioners who are working in communities to achieve sustainable behaviour change. To be of more practical assistance for future implementation, we intend to engage NGOs and other actors through global health conferences and other venues for exchanging practical methods for improving health interventions.

These dissemination efforts will be more thoroughly planned during the first year of the project and will ultimately result in the development of a ‘toolkit’ for practitioners to use in network-based intervention efforts. This toolkit will provide guidelines for collecting network data; develop open-source software to collect network data and identify network targets in field settings; and develop network data-collection materials suited to project goals and social context.
