# Supplementary material for: Exploiting social influence to magnify population-level behaviour change in maternal and child health: study protocol for a randomised controlled trial of network targeting algorithms in rural Honduras
Source: BMJ Open. 2017 Mar 10;7(3):e012996. doi: 10.1136/bmjopen-2016-012996 (PMC5353315; doi:10.1136/bmjopen-2016-012996)
Supplement: supplementary appendix [file bmjopen-2016-012996supp_appendix1.pdf]

Supplementary Appendix 1 for Exploiting Social Influence to Magnify Population-Level Behavior Change in Maternal and Child Health: Study Protocol for a Randomized Control Trial of Network Targeting Algorithms in Rural Honduras

Distribution of village populations and expected eligibility:

Our initial census estimates show approximately 32,500 people within the study population of 176 villages. Of these, approximately 11,495 (or 38%) are women between the ages of 15-49, 8% of whom (~920) may have a birth event during the period of our intervention (based upon age specific fertility rates available for the Copan region of Honduras on the Demographic and Health Survey Statcompiler). Final intervention enrollment numbers will depend on exact sampling strata.

Social network measures:

Social network analysis can provide several distinct measures that together allow researchers a comprehensive vantage point from which to study health behavior. The building blocks of social networks are individuals and their connections ("nodes" and "edges" or "ties"). In analysis, individuals under study are termed "egos" and their identified social connections are termed "alters" (see SA Figure 1, panel A).

Network level measures

In this study, we will be collecting sociocentric network data, and so we will be able to examine structural characteristics of entire villages and the individuals within them. For example, we can identify natural communities in the network ("cliques") that comprise groups of people who are connected together through relatively strong groupings of ties (e.g., SA Figure 1, panel D). We may find that certain clusters of women localized within particular regions of the network resist or adopt intervention uptake. We can also measure network *density*, which is the proportion of all possible two-person connections that are actually present. We may find that intervention adoption is more easily facilitated in higher density villages. Analysis of structural network characteristics like these will provide us with powerful tools for future interventions by allowing us to (1) predict what areas of the network will be most resistant or amenable to change, (2) leverage existing resources to target the population most effectively using network characteristics, and (3) understand possible changes in network structure that can either hinder or support intervention diffusion.

Individual-level measures

In addition to network-wide metrics, we will also examine individual network characteristics. The most frequently measured individual-level network metric is centrality, which is relatively intuitive in concept and is a measure of how "central" an individual is within any given network. In these analyses, we will be able to investigate to what degree individual network centrality can affect the adoption of the intervention components by any individual within the population. For example: (1) Do participants with higher centrality adopt the new intervention more readily than those with lower centrality? (2) Do individuals connected to high centrality social contacts adopt the new intervention more readily? (3) Are socially isolated individuals less likely to adopt the new intervention? (4) Is the relationship between intervention adoption and education moderated by network characteristics (e.g., will educated women who are less socially connected adopt the intervention less readily than educated women who are well connected)?

Centrality can be operationalized in a number of ways. Each operationalization holds the similar feature that the score assigned to the individual increases when the individual is more central under the particular objective function. For example, one centrality measure aims to identify individuals who are key “hubs” in the network through which information must travel to get from place to place; another centrality measure aims to identify individuals who have a large number of well-connected friends[1]. Although distinct centrality measures are built to identify distinct features of the position of the individual, in practice, each of the measures are correlated with one another.

Several centrality measures have been shown to perform well in identifying important individuals in social[2] and epidemiological networks[3]. The basis of most centrality measures is “degree”, or the total number of ties that an individual holds. *Degree centrality*[4] then, is simply the total number of unique social connections that nominate or are nominated by the subject (SA Figure 1, panel B). Degree is often referred to as a first-order metric, since it simply counts the number of ties connected to each individual; degree has the desirable property of being rank-unbiased in a sampled network[5]; however, it degree does not provide any information about an individuals' more general location in the network. Further refinement of this measure might generate scores based only on social nominations received (*in-degree centrality*) or sent (*out-degree centrality*).

Other measures of centrality look beyond direct ties to provide a fuller measure of where an individual ties within in the larger network. It is possible to measure the social distance between any pair of individuals in the network by defining one's friends to be at distance 1, the friends of one's friends at distance 2, and so on. *Closeness centrality*[6] is the inverse of the average distance between a respondent and all other people in the network[7]. A third measure, *betweenness centrality*[8], identifies the extent to which an individual in the network is critical for passing support from one individual to another while a fourth measure, *eigenvector centrality*[9], assumes that the centrality of a given individual is an increasing function of the centralities of all the individuals that support her.

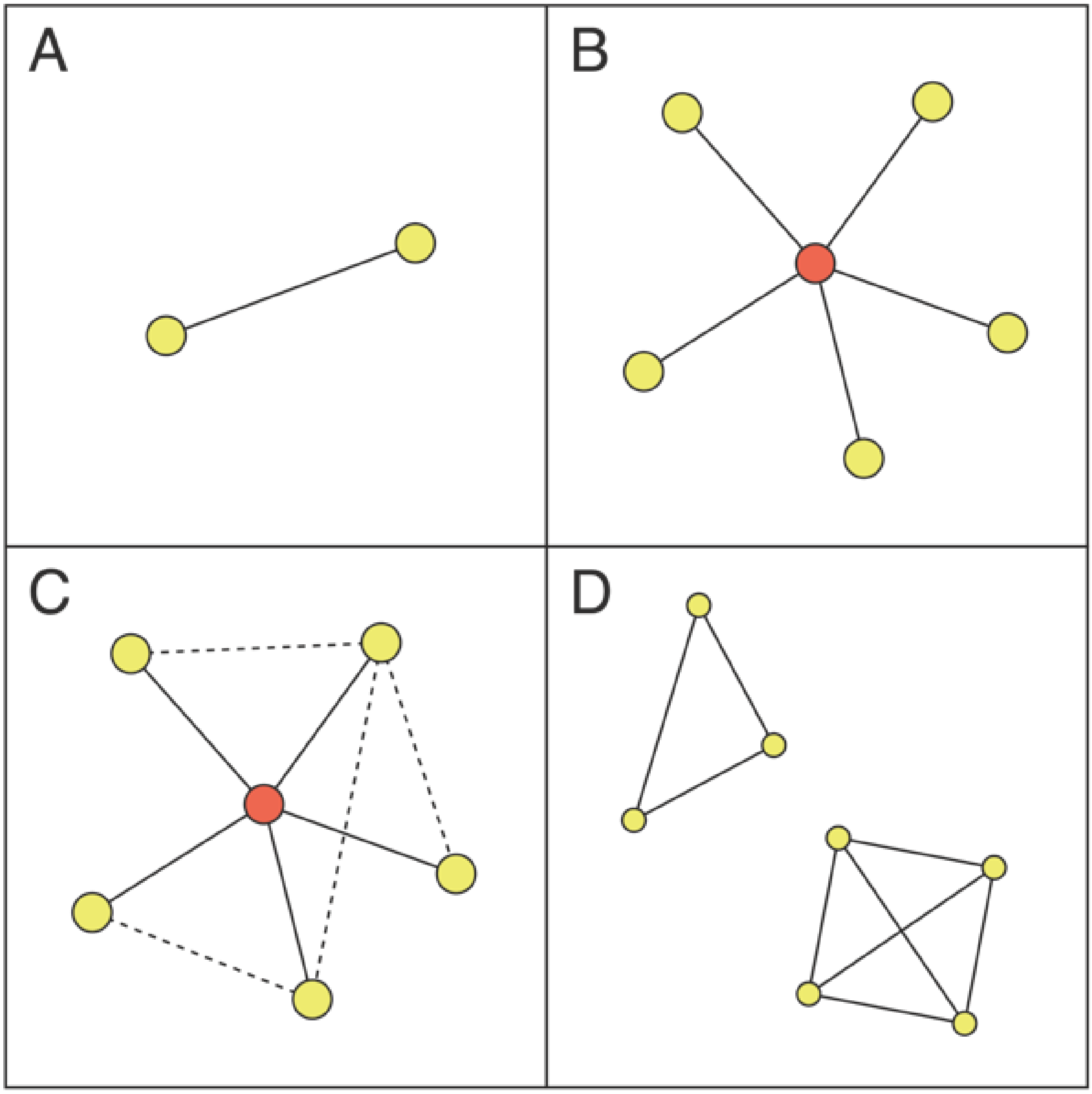

SA Figure 1: *A schematic illustrating some fundamental concepts about social networks. (A) Nodes and ties are the elementary building blocks of networks. A tie connecting two nodes indicates a social relationship between the two individuals. (B) The degree of a node (sometimes called "degree centrality") is a metric that quantifies the number of connections (acquaintances, friends, etc.) a given node has. For example, the red node at the center of the figure has a degree of five. (C) The clustering coefficient is a metric that quantifies the extent to which the network neighbors of a given node are directly connected to one another. More specifically, the clustering coefficient of the individual at the center of panel C (red node) is given by the number of ties that exist among his or her friends (the dashed four ties) divided by the number of ties that could exist between them (in this case, 10), yielding a value of 4/10 or 0.4. This number can also be interpreted as the probability that any two randomly chosen network neighbors of an individual are connected. (D) Social networks typically possess meaningful structure beyond the level of nodes and ties. So-called "cliques" are typical examples of such structure, and panel D exemplifies a 3-clique (top) and a 4-clique (bottom), which consist of three and four nodes, respectively, and the realization of all possible ties between them.*

Pilot work

Prior to the rollout of our community census and baseline survey efforts, we conducted 3 separate pilot surveys. The first pilot survey, conducted in the summer of 2014, was given to 1018 individuals in 2 towns in the department of Lempira, Honduras, of whom we were able to collect network, demographic, and social normative measures on 831. The second pilot survey was conducted in February 2015, and covered 4 small villages in the Copan region, and included 165 individuals with questions on networks, demographics, as well as reproductive, maternal, child and neonatal health (RMNCH) knowledge, attitudes, norms, and behaviors. Our final pilot survey was conducted in May 2015, for which we enrolled 577 individuals. After each pilot we carefully analyzed the data for anomalies, including large number of missing responses, no variation in item responses, or responses that seemed irregular in ways suggesting response bias or misunderstanding of questions. The survey was then revised accordingly.

Field operations

We recruited and trained over 100 local surveyors to perform preliminary data collection and infrastructure development, complete participant recruitment and census enumeration, and conduct the survey interviews. We also established 3 field offices in geographically strategic locations to minimize travel time to and from the study villages. All offices are fully equipped with data collection tablets, netbook computers, printers, high speed internet, and a local server to insure secure download, synchronization and transfer of research data. All surveyors were extensively trained in the use of our survey instruments and software by US-based project managers and supervised by Honduran project coordinators with whom we have daily contact in relation to data collection and other implementation activities.

Community engagement

We developed valuable relationships with local government and health officials, participating in staff meetings for health center personnel and community health workers. We presented project plans and obtained approval of our field operations and data collection procedures from the Honduras Ministry of Health (MOH). The MOH has reviewed our study protocols, consents and survey instruments and provided feedback. Our field teams have also met with local community leaders and indigenous council members to present study objectives in all study villages prior to beginning recruitment and data collection.

Registration

This trial is registered with ClinicalTrials.gov, number NCT02694679.

Data collection

Geographic Maps

The first stage in data collection is making geographical maps of each village. The maps serve as a means of quality control and help focus the efforts of each survey team. SA Figure 2 shows a map from a Los Ranchos, a village in our pilot study. The village of Los Ranchos is typical of the size and makeup of communities in this study's sample. The mapmakers use satellite imagery to get an idea of the town's layout, after which the team goes into to the village and place every edifice on the layout. Once the map is completed every structure is given a unique code that enumerators will use to when conducting census and surveys

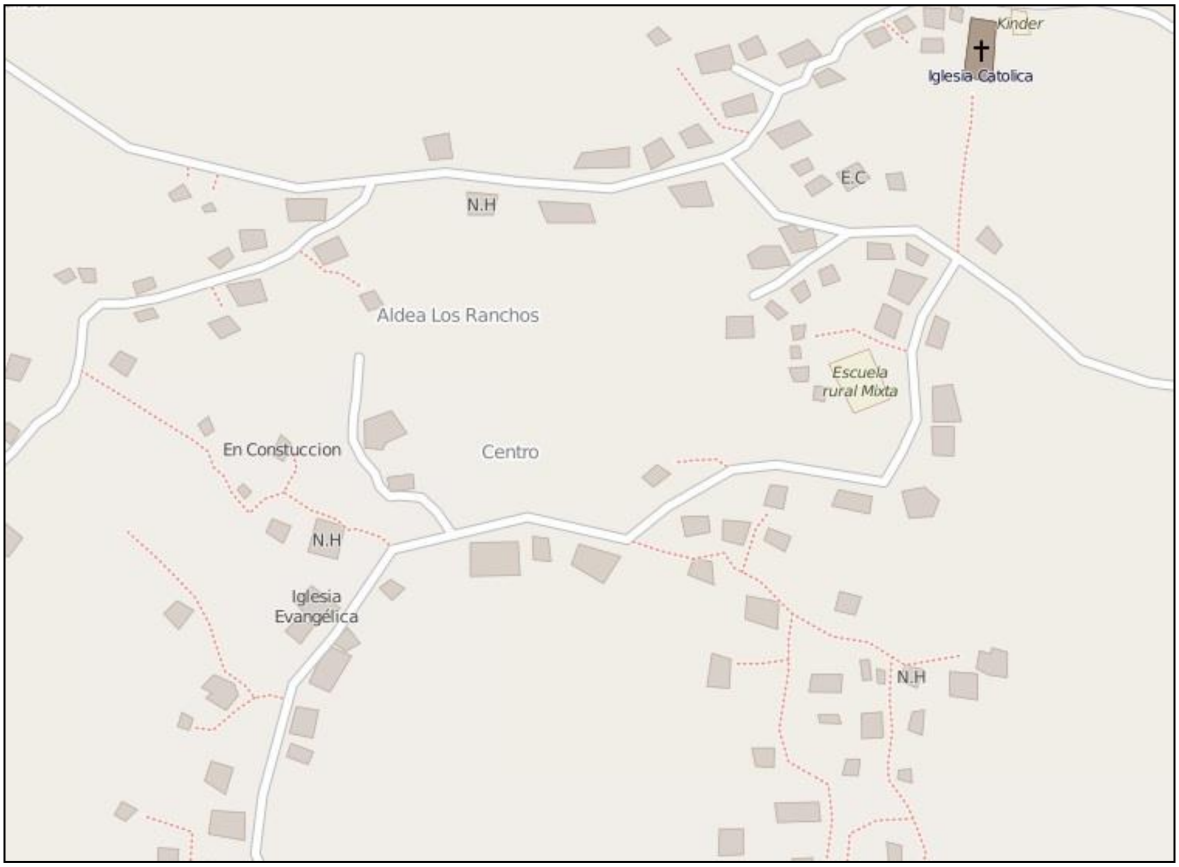

SA Figure 2. *Map of Aldea Los Ranchos, a village in Copan. All dwellings and roads added and confirmed by our study surveyors are now available for public use on www.OpenStreetMaps.org*

Photographic Census

After the map of a town is complete, enumerators will visit each building and ask to photograph each person living in the residence. These pictures will be taken on tablets preloaded with *Trellis*, a tablet-based survey program designed to facilitate network data collection in the field. The pictures and some preliminary demographic information will be saved to the program's database so the pictures can be used in the next stage to increase the accuracy and efficiency of network data collection. We use photo identification primarily because to solve issues of unique identification in the presence of name similarity and high illiteracy. During previous data collections efforts in this area, the research team learned that in one town fifteen women were named Maria Hernandez. And so, when a respondent answers that Maria Hernandez is one of her friends, enumerators would have been stymied without additional information to specifically identify one Maria from the others. Past efforts have measured the additional information by asking qualifying questions about Maria; however this increases the time it takes to gather data while decreasing accuracy. Pictures are faster and more accurate.

Large Survey and Network Data Collection

Upon completing the photographic census, we will undertake the baseline survey in each village. This survey includes the battery of “name generators” that serve to identify the social alters, thereby building each villages’ social network[10]. Name generators will cover a broad grouping of relationship types, including affective, kinship, and resource exchange. To uniquely identify the connections between individuals, we will use the photographs collected in the census.

Name generator questions

Does your mother live in this town?

1.) If yes, what is the name of your mother?

Does your father live in this town?

2.) If yes, what is the name of your father?

What are the names of your siblings over the age of 12 that live or work here?

Do you have any children who don’t live with you but do live in this village over the age of 12?

3.) What are their names?

Are you married or living in a civil union?

4.) What is the name of your partner?

In the next section, we will ask you some questions about who are the people that you do different things with, and to name those people specifically. You can answer the questions with names of people who are OVER THE AGE OF 12 and WHO CURRENTLY LIVE OR WORK IN THIS VILLAGE ONLY. These people may include any friends, family, people you work with, people that work for you, neighbors, etc. When you answer these questions, you may answer with one person, more than one person, or say there is no one. You are free to include up to 5 people.

5.) Who do you trust to talk to about something personal or private? (including friends, family, people you work with, neighbors etc, who live or work in this village)

6.) With whom do you spend free time? (including friends, family, people you work with, neighbors etc, who live or work in this village)

7.) From whom would you feel comfortable asking to borrow 200 Lempiras if you needed them for the day? (including friends, family, people you work with, neighbors etc, who live or work in this village)

8.) Who do you think would be comfortable asking you to borrow 200 Lempiras for the day? (including friends, family, people you work with, neighbors etc, who live or work in this village)

9.) Who would you ask for advice about health related matters? (including friends, family, people you work with, neighbors etc, who live or work in this village)

10.) Who comes to you for health advice? (including friends, family, people you work with, neighbors etc, who live or work in this village)

11.) Besides your partner, parents or siblings, who do you consider to be your closest friends? (including friends, family, people you work with, neighbors etc, who live or work in this village)

12.) What are the names of the towns leaders?

We have asked questions about the people who are positive connections in your life. Our study aims to group people that get along well. Now we are going to ask you one question about the people in your life with whom you usually do not get along well. It is important that you understand that we will keep all of your responses secret and that they will only be used for the purpose of mathematical analysis. Your information will never be shared with anyone.

13.) Who are the people in this town with whom you do not get along well? (including friends, family, people you work with, neighbors etc, who live or work in this village)

Do you have a patron/patrona?

14.) What is his/her name?

Participant attrition and missing data:

Over 93% of community residents have agreed to participate in the study, so bias due to failure to recruit participants is likely to be very low. Observations with missing data will be dropped from the analyses, which will also not create bias in the data as assignment to treatment groups, and therefore missingness, is random. We will also make our best effort to keep track of study participants who move from one study village to another, in order to retain them in the study.

Research Design

Assignment of villages to treatment arms in the 8x2 design.

We ran a procedure that generates 10,000 assignments of villages to treatment arms. This procedure generates assignments while controlling balance on the following variables.

Number of respondents in the village
Average number of respondents in the household
Latitude
Longitude
Elevation
Time to health center
Time to maternity clinic

This procedure prioritizes matching the means and standard deviations of 16 treatment arms, but also weights the third moments and cross-moments (to get at covariances). The procedure also assigns a score that summarizes balance.

We checked how similar these assignments are. Of the 100 assignments with a better balance summary score, no two contained a pair of treatment arms that overlap in more than 8 elements. We also measured the average maximum overlap, defined as the maximum number of items that any pair of treatment arms have in common. On average, over the 100 assignments with better balance summary score, the maximum overlap was 3.985. So, the top 100 assignments of villages to treatment arms were are all quite different.

Focusing on the 100 assignments with better balance summary score, as candidate re-randomizations, we then ran a battery of statistical tests check for statistically significant imbalances. We focused on the following variables and tests quoted in parenthesis.

Number of respondents in the village (t test)
Average number of respondents in the household *(t test)*
Latitude (t test)
Longitude (t test)
Elevation (t test)
Time to health center (t test)
Time to maternity clinic (t test)
Number of households (t test)
Empirical distribution of household sizes (Kolmogorov-Smirnov test)
Village can be accessed in when raining (proportions)
Proportion of indigenous population (t test)
Time in minutes to main road (t test)
Village is a coffee producer (proportions)
Average number of women of reproductive age (t test)
Average age (t test)
Percent male (t test)

For each of the 100 assignments we produced a table with these tests for each of 147 contrasts: 120 contrasts reported the p-values for imbalance in pairs of treatment arms for each of the covariates; 26 contrasts reported the p-values for marginal imbalance in pairs of levels of treatment; 1 contrast reported the p-values for marginal imbalance in the two nomination schemes.

We selected assignment number 73, which had only one imbalance at significance 5%. The imbalance was for average age between treatment arms 3 (random nomination, 10% treated households) and 14 (friend nomination, 50% treated households), which is an unimportant contrast for the purpose of conducting the analyses proposed in this study.

Assignment of households to treatment/control in each village.

Treatment is to be understood as percentage of households, which was applied to the number of households in each village and the rounded to the nearest whole integer number.

We built a network where a tie meant that there was a tie in any of the following name generators: personal private matters; spend free time; closest friend.

For each village, we generated 10,000 assignments of households to treatment in villages in the friend nomination arm. For each of these assignments we then computed balance for the following covariates.

Number of census respondents
Number of women of reproductive age
Number of children under the age of 12 who live in this household
Existence of handwashing location observed by enumerator
Household electricity
Separate room in the house that is used as the kitchen
Self-reported health status
Network degree centrality of the household

We also considered the following individual variables averaged at the household level.

Age
Sex
Baseline self-reported health status of the individuals in the households

Here, for each of the 144 villages assigned to a treatment arm in which the percentage of household treated differs from 0% and 100%, we only considered the contrast: treatment vs. control.
We then selected the set of assignments of household to treatment and control in each of the 144 villages that minimized the number of tests for imbalance failed at 5%.These balanced sets of assignments constituted the final assignments of households to treatment in villages in the random nomination arm, but only the initial assignment of households to treatment in villages in the friend nomination arm. For villages in the friend nomination arm, we picked each of the households initially assigned to treatment, in turn, then picked a person in the household, then picked a tie at random among this person's ties. Whenever we ran into a duplicate, we restarted the choice. This procedure generated a final set of assignments of households to treatment in villages in the friend nomination arm.

Statistical methods and details of analysis plan

Analytic Aim 1: Evaluate the Extent to which Behavior Change Regarding RMNCH Care Spreads
For this analytic aim, we will use the results of the RCT to ascertain the extent to which beneficial or harmful RMNCH *attitudes* and *behavior change* in one person can influence the likelihood of attitudes and behavior in other people to whom they are connected. The success of this aim will allow us to develop methods by which we can target specific individuals in future interventions to disseminate RMNCH care interventions in order to achieve the greatest uptake at the lowest cost. To test whether attitudinal or behavioral changes related to the outcome variables diffuse, we will measure the *indirect effect* of the treatment on the *friends* of the treated. We can do this with the following model:

$$E(y_j) = \alpha + \beta\tau_j + \lambda\Sigma_k a_{jk} \tau_k \qquad (1)$$

where y_j is a binary variable indicating whether subject j engages in the outcome behavior (e.g., skin-to-skin warming or following dry cord care protocol); τ_j indicates whether subject j was treated; β is the direct treatment effect; a_{jk} indicates if subject j names subject k as a friend; τ_k indicates whether subject k was treated; and λ is the indirect treatment effect that estimates the effect on subject j of having an additional friend who was treated. We will test the hypothesis that $\lambda = 0$. If $\lambda > 0$ then it means the treatment is spreading to other people.

Note that τ_k is randomly assigned, but $\Sigma_k a_{jk}$ is not (it is simply the total number of friends subject j has). Therefore, to preserve pure randomization, we have to condition model (1) on $\Sigma_k a_{jk}$ by running separate regressions for all individuals with the same number of friends (one model for all subjects who have 1 friend, another model for all subjects who have 2 friends, and so on). We then combine the coefficients from the separate regressions, weighting them according to the number of observations in each regression and adjusting the standard errors accordingly (this is similar to using meta analysis to combine effect sizes estimated from separate randomized controlled trials). To estimate the model we will use ordinary least squares, but to check for robustness we will also use a logit specification.

Specific questions we will be able to answer through these methods include: (1) Is there an inter-individual spillover effect for the intervention? (2) Does social influence of one's social contact facilitate faster or more effective adoption of the intervention? (3) Does influence regarding RMNCH care practices spread not only from mother to mother, but from mother-in-law to mother-in-law or husband to husband? (4) Are there covariate moderators to possible social influence effects, such as income, age, or education? (5) Is there an average number of degrees of separation (the number of steps in the network it takes to get from one person to another) at which the path of influence fades?

We will explore a variety of model specifications during our analysis phase, and conduct diverse robustness checks when the independent variable is not randomly assigned. We can evaluate the possibility of omitted variables or confounding events explaining the associations by examining how the type or direction of the social relationship between ego and alter affects the association between ego and alter (using a "network directionality" test)[11]. If unobserved factors drive the association between ego and alter, then directionality of friendship should not be relevant; the outcome variable in the ego and the alter will move up and down together in response to the unobserved factors. In contrast, if an ego names an alter as a friend but the alter does not reciprocate, then a causal relationship would suggest that the alter would significantly influence the ego, but the ego would not necessarily influence the alter.

To further test the robustness of the results, we will experiment with different error specifications, such as Huber-White sandwich estimates with clustering on the egos. We will also test for the presence of serial correlation in all GEE models using a Lagrange multiplier test[12]. Additionally, we will also conduct a sensitivity analysis specifically recommended by Vanderweele [13] in which we estimate the bias in the association between ego and alter that might be caused by an omitted variable that is correlated with the prevalence of the outcome variable in both ego and alter. This class of omitted variables includes those explain friendship formation based on the trait (homophily) and those environmental factors that could affect ego and alter independent of their relationship (confounding). These sensitivity analyses show how the association changes given differences in prevalence of an omitted variable that are conditional on the alter's trait and given the size of the effect of the omitted variable[13]. In this way we can evaluate how likely it is that an inference from observational data depends on something we have not measured. Of course, our experimental study is relatively unaffected by such concerns.

Analytic Aim 2: Test for Social Effects

One of the primary goals of this project is to understand the ways in which social network strategies can be used to maximize the population-level impact of interventions. By understanding and anticipating social effects, we can direct intervention resources so that they achieve the maximum impact per dollar invested.

Suppose we imagine an intervention in which there is no social effect (the "No Social Effect" red dashed line shown in **Figure 5**). In this situation, we would expect there to be a proportional increase in the number of people who change their behavior in response to the number of people targeted. So, perhaps when we target 20% of the population, 10% actually adopt; when we target 30% of the population, 15% actually adopt, and so on. In other words, there is a linear relationship, and the rate of adoption is directly proportional to the rate of initial exposure. In this scenario, no matter how many people we target, the *rate* of adoption will remain constant. We get just as much return on our investment when we target the first person as we do when we target the last. In contrast, if there are social effects, the relationship between percent treated and percent adopting will be *nonlinear* (the "Social Effect" blue dotted line shown in **Figure 5**). By social effects, we simply mean that people will tend to be influenced by the behavior of their social contacts. We hypothesize that, initially, this effect will be negative. When an intervention is unfamiliar to a community, people may discourage potential adopters, so that the rate of adoption will be lower than the "no social effect" line. However, we anticipate that once the intervention reaches a certain level of saturation enough adopters will exist in the population that people will stop being discouraged from adoption. Moreover, at some level, some untreated subjects will copy the behavior of their social contacts who have adopted. As more people adopt, the overall community attitude towards the intervention becomes more encouraging, and the positive cycle that ensues will create higher rates of adoption for every person targeted. Once most of the people who are likely to adopt have done so, however, the rate of adoption slows again, creating the classic S-shaped adoption curve shown in **Figure 5**.

A key aim of this study is to test whether or not we observe S-shaped adoption curves in the various behaviors that we target. Our RCT plan calls for varying the proportion of individuals that are exposed to the intervention at the town level. This variation allows us to test the difference in the rate of adoption at these various treatment proportions in a way that is un-confounded by the spillover that we aim to leverage. The null hypothesis is that adoption is a linear function of the number of people treated across villages that are randomly assigned to different treatment regimes:

$$y_i = \alpha + \beta x_i + \Delta t_i + \varepsilon_i \qquad (2)$$

where y_i is the outcome variable (such as early initiation of breastfeeding, use of thermal care, facility births, etc.) for village i; α is the baseline rate of adoption; x_i is the randomly-assigned proportion of individuals in the community that are treated; β is an estimate of the full treatment effect when all individuals in the village are treated; t_i is a binary variable that indicates whether the village has been randomly assigned to receive the friend-targeting treatment, and Δ is an estimate of the difference between friend targeting and random targeting.

In model (2), a test of $\beta = 0$ can be used to evaluate whether there is a significant effect of the treatment on the outcome. A test of $\Delta = 0$ can be used to evaluate whether friend targeting is more effective than random targeting. But this model does not test our hypothesis about social reinforcement because it assumes that the treatment effect is constant regardless of the fraction of the village treated. We therefore introduce an alternative model:

$$y_i = \alpha + \beta\,(1 + \exp(\,-(x_i + \Delta\, t_i - \theta) / \sigma)\,)^{-1} + \varepsilon_i \qquad (3)$$

Just like model (2), model (3) can be used to test the treatment effect (β) and the difference between friend targeting and random targeting (Δ). However, model (3) includes two extra parameters that allow us to test whether social reinforcement is present. The shape parameter σ indicates the strength of social reinforcement (lower σ yields more curvature towards 0 for low values of x_i and more curvature towards 1 for high values of x_i, indicating it is harder to get people to change when few people are treated and easier when many people are treated). The location parameter θ indicates the critical threshold where social reinforcement *in favor* of the behavior change takes over social reinforcement *against* the behavior. These two parameters will allow us to learn what fraction of a village can be treated in order to achieve nearly the same effect as treating the whole village.

To test whether social reinforcement is present, we can use nonlinear least squares to fit both models and conduct an ANOVA *F*-test. Note that this test requires the models to be nested and in this case they are since model (3) converges to a linear model just like model (2) as σ becomes large. If model (3) fits the data significantly better than model (2), then it suggests that social reinforcement is present. Detection of social reinforcement requires a 2 x 8 design because we need additional precision in the region between $x_i = 0$ and $x_i = 0.5$ to estimate the curvature away from the linear model. And we have chosen more cells where x_i is less than 0.5 because anecdotal evidence suggests that the critical threshold is in that region – some practices can become popular once just a few people adopt them.

Analytic Aim 3: Test the impact of the "nomination" network targeting method
In this aim, we attempt to ascertain whether it is possible to reduce the total percentage of people we need to treat in order to achieve the same effect.

Nomination targeting is the practice whereby an individual is chosen at random from the population, and then a random friend of that individual is targeted to receive the intervention packet. This targeting leads to intervening with individuals who have more social connections, and as a result are connected o more people in the network, and are therefore potentially more influential than individuals chosen at random. Previous work suggests that the adoption curve under nomination targeting is shifted to the left relative to random targeting because friends are more central in the network and therefore spread information and norms more quickly in the early part of the intervention. This shifts the whole adoption curve to the left, as shown in **Figure 5** (the "Enhanced Social Effect" shown by the solid dark blue line). As a result, the total percentage of individuals that need treatment to achieve nearly-maximum adoption also shifts to the left (in the example, it shifts from about 60% to 40%), meaning that we can treat even fewer people to achieve the same desired effect within each village.

We can explicitly test whether there is a shift in the adoption curve with the shift variable, Δ, noted in equations (1) and (2) shown above. A significant coefficient would suggest that friend targeting achieves significantly more efficient results than random targeting for a given health behavior (without increasing the number of people targeted or the expense involved). And, if so, it also suggests that it might be possible to apply this method to behavioral intervention targeting strategies in a variety of different contexts in order to maximize intervention uptake in many global settings.

Analytic Aim 4: Evaluate the Impact of Network Structural Characteristics on Behavior Change
Descriptive characteristics will be calculated for the network at the village level. Subgroups such as cliques, components, and communities will be identified within these village networks (in particular, using state-of-the-art community detection algorithms that have recently been invented [14]). These subgroups will be identified in two different ways. We will identify subgroups based solely on network properties, independent of any covariate properties; and we will also identify subgroups based upon correlated values of covariates, such as education, income, or gender norms.

Discovery of characteristic-based subgroups involves testing the assumption that individuals with strong network ties will be more likely to share common attributes, and that the stronger the network tie (i.e., the closer to each other they are within the network), the higher the correlation between attributes. While there is no unique method for characterizing this network correlation structure, we can build on methods from spatial statistics, making suitable modifications to adapt to the complexities of network topology.

Mean values of covariate variables within each relevant subgroup will be calculated and compared using regression models to determine whether these differ across groups. We will create additional variables for each individual participant for subgroup membership and regress our outcomes of interest, including adoption of the intervention and rate of adoption, against these predictors. With this type of modeling, we might address descriptive questions such as: (1) for which health behaviors or demographic characteristics are network correlations stronger or weaker; (2) are correlations based upon certain types of attributes (such as education or wealth) more predictive of RMNCH care behavior than others; or (3) are covariate-based subgroups more predictive of intervention uptake than covariate-independent subgroups?

Individual-level network characteristics, such as centrality and transitivity, will be calculated for each participant in the dataset. We will test whether these characteristics are predictive of individual intervention adoption as well as overall subgroup intervention adoption (i.e., do differing mean values of individual network characteristics at the subgroup level predict different rates of intervention adoption). Using the general framework in equation (3), we will then test whether these network characteristics are moderators of the relationship between important predictors, such as education and income, and our outcomes of interest.

Results of these analyses will include: (1) network measures for all individuals within the network and higher-order features like clusters and communities for the overall network itself; (2) results showing the relationship between network measures and outcomes of interest, including any possibly moderating effects; (3) recommendations for utilizing network measures, including network position and network subgroups, for future interventions

Analytic Aim 5: Ascertain Whether Partial Collection of Network Data Can Help Identify the Most Influential or the Most Influence-able People in the Network

One of the main hypotheses of the RCT of network targeting using our intervention is that the nomination technique will help us to quickly identify the most central people, and that this means of identification will allow us to diffuse the intervention more effectively. However, another important question we will address is what name generator to use to identify the nominated friends in the future. Is *friendship* the most important relationship to identify in order to find those who are most influential with respect to RMNCH care behaviors, or do name generators need to be specific to the context?[10] In other words, a person who is most socially central to the community may or may not be the most central in terms of health behavior opinions. Hence, we will analyze which name generators uncover the relationships most influential to our RCT behaviors. These analyses will inform a deeper understanding of the utility of name generators in identifying the most influential people depending upon the context.

The structure of networks can also greatly influence adoption. Depending on the behavior in question, a tightly knit community may resist changing norms, but it may change swiftly once such norms begin to be established. By applying network simulations to our network data, we will develop strategies for estimating optimal targeting given the observed rates of behavior transmission and the measured density and transitivity of the network. We will then experiment with sampling techniques that would allow for choosing which strategies work best when there is only partial network information.

Network centrality is intuitively appealing in terms of disseminating interventions. It makes sense that the people who are most "central" to a community may be those whose opinions have the most exposure within that community. If one wins those people over, then new behaviors and attitudes are likely to spread. However, this also assumes that those most central may be the most amenable to accepting the new behavior of interest. There is evidence that this may not always be the case. In densely connected communities where norms are tightly held within communities, *the most central individuals might be the most resistant to change*[15]. This may particularly be the case where the behavior in question challenges a strongly held norm, and where the community conspicuously sanctions transgressions. Central individuals may pay the highest cost for infractions, as their conformity to the norm is a matter of great interest to those around them.

Our work on the adoption of latrines in India suggests that the most central may resist adoption, and are potentially less amenable to change than those on the periphery of the network[15]. In a situation such as this, initially infusing the network from the periphery may make more sense than beginning at the center. On the other hand, interventions that use the center of the network as the point of infusion may find that the most peripheral to the network have not been engaged in the intervention. In this case, those people more peripheral to the center of the network, who were potentially the most marginalized to begin with, may become even more marginalized as a result of not being reached by the intervention. Our analyses will allow us to explore this too, and to develop guidelines for which kinds of interventions and which kinds of contexts benefit most from targeting the core versus the periphery of a network.

Analytic Aim 6: Assess the effect of the intervention on the village social networks

To assess the direct effect of treatment on individuals' local networks in the study, we will measure individual level network statistics (degree centrality, transitivity, closeness centrality, betweenness, and eigenvector centrality) at the end of the study and use a simple difference of means test to evaluate whether these metrics vary between subjects assigned to treatment and those that were not assigned to treatment. We will additionally regress network statistics on an indicator for whether the subject received treatment and network variables measured at baseline to improve efficiency in the comparison, and we will cluster standard errors by village to be sure that between village effects do not interfere with the estimation of the individual level effect.

To assess the indirect effect of treatment, we will use model (1) to estimate the effect of a friend's treatment on the subject's network measures as noted above. For example, if a friend receives treatment does that increase the number of the subject's social contacts within the village?
We will additionally measure village level network characteristics (mean degree, variance in degree, and mean transitivity) and assess the extent to which treatment assignments influenced these outcomes using model (2) as noted above. To improve efficiency, we can also include as an independent variable the village level network measure as assessed at baseline.

Power Calculations
We have conducted extensive power calculations on all 3 models shown above. In our core simulations, we assume that individuals in each village are exposed to the randomized treatments proposed for each village and the "true" probability of an individual outcome is generated by models (1), (2), or (3). We then used those probabilities to draw a Bernoulli random outcome for each individual based on their assigned probability.

Once we generated the "true" data, we then fit the corresponding model to it to see if the model generated significant hypothesis tests for a given set of parameters. We repeated this process 1000 times to estimate the percent of the time the model was able to identify significant results.
For models (2) and (3), we also explored whether model misspecification affected power (we used model (1) to generate the data and model (3) to recover it, and vice versa) and found that a two-stage approach works best. We fit both models to the data, and if there is evidence that model (2) fits better using the ANOVA test, we then use the estimates from that model. Otherwise we use the estimates from model (2).

In our core simulations, we assume that the baseline rate of the outcome is $\alpha = 0.2$. Across all simulations, most assumptions of the baseline rate yield identical results (only values near 1 potentially change the results because of ceiling effects on the possible size of the treatment), so this assumption is not critical. For simulations where we are not evaluating their power, we assume that the full treatment effect $\beta = 0.1$, and the difference between friend targeting and random targeting $\Delta = 0.1$.
These assumptions are conservative compared to the known treatment effects of the interventions we are evaluating here. For example, in our own pilot, we find that friend targeting can increase uptake of a nutritional intervention by 12.2%, compared to control populations[16]. They are also conservative relative to the power of the design, since below we show that we have more than enough power to identify effects of those magnitudes.

For simulations from model (3) we also assume that the shape parameter indicating the strength of social reinforcement $\sigma = 0.1$, and $\theta = 0.25$, which yields moderate curvature of a maximum about 0.2β away from the linear model when the percent of the village treated is $x_i = 0.25$. Little is known about what the right assumptions are here, but values of $\sigma > 0.2$ are approximately linear given the range of treatment effects for β that we tested, and alternative simulations that assume $\theta = 0.5$ yield similar results.
Finally, for model (1), we assume that the social connections a_{jk} are distributed as a random network with mean degree $\Sigma_k a_{jk} = 5$ (in other words, the average person has 5 randomly chosen friends). In alternative simulations utilizing small world and scale free networks, the results are identical, suggesting that the degree distribution and clustering in the network are not critical.

Figure SA 3 shows the main results of our power tests.

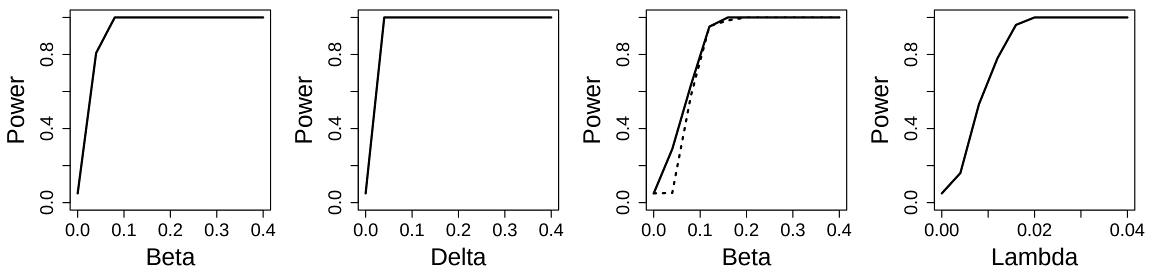

SA Figure 3: *The first panel shows that when we use our core simulation assumptions and vary the full treatment effect (β), we can detect values of β > 0.05 at least 80% of the time. Similarly, the second panel shows that when we vary the difference between random and friend targeting (Δ), we can detect values of Δ > 0.04 at least 80% of the time. In the third panel, we simulate data from model (2) and use the ANOVA F-test to evaluate whether model (3) fits better than model (2) to test for the presence of social reinforcement. The results show that even when the full treatment effect is quite low, we can still detect social reinforcement 80% of the time when β > 0.12. The solid line shows results for a 2 x 8 design, while the dashed line shows results for a 2 x 5 design. The 2 x 8 design shows slightly better power when β is small. In all other power analyses, there was no difference. Finally, in the fourth panel, we test model (1) and show that we can use within-village random variation in percent treated to detect the indirect effect on a subject of having an additional friend who was treated, even when it is very small (λ > 0.01). This value suggests we can identify a total indirect treatment effect for someone with 5 friends of about 0.05, which represents a "friend multiplier" of about 0.5 the size of the direct treatment effect. For comparison, note that total indirect effects are typically much larger than that, estimated to be about 1.7 for emotional contagion, 3.0 for public goods provision, and 4 to 5 for transmission of voting behavior .*

Evaluation of Model Specification

This is a randomized controlled trial, so the primary concern is not inclusion of control variables. Appropriate control variables can improve efficiency, but their exclusion does not bias the results as long as they are uncorrelated with the treatment. Therefore, the biggest threat to the research design is so-called "randomization failure." As with all our previous experimental studies, we plan to conduct thorough balance tests to establish whether or not – by chance – there is a relationship with assignment to treatment and village features such as population size, baseline outcome incidence prior to the intervention, and demographic factors. For model (1) our balance tests will also include outcome measures and demographic factors of friends to ensure that the friends of the treated have the same distribution of key variables prior to the experiment as the friends of the controls.

Another concern is model misspecification. We address this above by identifying a strategy for distinguishing between the linear model (2) and the nonlinear model (3) specification. For model (1) one potential alternative assumption is that the percent of friends treated rather than the total number of friends treated is the key variable for transmission of the treatment effect. We can easily test this alternative by fitting the following model for comparison:

$$E(y_j) = \alpha + \beta\tau_j + \lambda\Sigma_k a_{jk} \tau_k / \Sigma_k a_{jk} \qquad (1b)$$

Additionally, there may be heterogeneous treatment effects, and we can test for these by interacting the treatment variable with variables that explain the heterogeneity. For example, we can test whether there is a relationship between the sex of the friend and the indirect effect in model (1) to see if women are better at transmitting effects to friends. Similarly, we can test whether the sex of the subject influenced the indirect effect (are women more *susceptible* to indirect effects?)

For all models, we will study histograms of residuals to assess their normality and conduct Shapiro -Wilk tests. Non-normality suggests that we many want to transform one of the variables in the model or to use a different model. For example, the outcome variables in models (2) and (3) vary between 0 and 1. If treatment effects are large relative to that range (specifically, if $\beta > 1 - \alpha$), there may be ceiling effects that would create downward bias in our estimate of the treatment effect. If so, we may want to use censored regression instead of ordinary regression. Similarly, the outcome variable in model (1) is binary and so we may want to use a logit model rather than OLS, though these models typically yield similar results when treatment effects are small.

Fitting nonlinear least squares models requires additional attention to residuals. We plan to study residual Sum of Squares (RSS) contours, which are similar to the likelihood contours for a Gaussian general linear model. In particular, we will identify the 95 percent Beale's confidence region in each plane of two parameters to explore potential multicollinearities and their effect on mo del estimates. Given potential nonlinearities in the profile likelihood, we also plan to verify 95% confidence regions of estimated parameters using non-parametric bootstrapping.

References

1. O'Malley AJ, Marsden PV. The Analysis of Social Networks. *Health Serv Outcomes Res Methodol* 2008; **8**(4): 222-69.
2. Freeman LC, Borgatti SP, White DR. Centrality in valued graphs: A measure of betweenness based on network flow. *Social Networks* 1991; **13**(2): 141-54.
3. Borgatti SP, Mehra A, Brass DJ, Labianca G. Network analysis in the social sciences. *science* 2009; **323**(5916): 892-5 %@ 0036-8075.
4. Proctor C, Loomis C. Analysis of Sociometric Data. In: Holland P, ed. Research Methods in Social Relations. New York: Dryden Press; 1951.
5. Kang U, Papadimitriou S, Sun J, Tong H. Centralities in Large Networks: Algorithms and Observations. SDM; 2011: SIAM; 2011. p. 119-30.
6. Sabidussi G. The centrality index of a graph. *Psychometrika* 1966; **31**(4): 581-603.
7. Lin N. Foundations of Social Research, New York: McGraw-Hill. 1976.
8. Freeman LC. A set of measures of centrality based on betweenness. *Sociometry* 1977: 35-41.
9. Bonacich P. Factoring and weighting approaches to status scores and clique identification. *Journal of Mathematical Sociology* 1972; **2**(1): 113-20.
10. Shakya HB, Christakis NA, Fowler JH. An exploratory comparison of name generator content: data from rural India. *Working Paper* 2015.
11. Christakis NA, Fowler JH. Social Contagion Theory: Examining Dyna mic Social Networks and Human Behavior. *Statistics in Medicine* 2012; **32**(4): 566-77.
12. Beck N. Time-series-cross-section data: What have we learned in the past few years? *Annual review of political science* 2001; **4**(1): 271-93.
13. VanderWeele TJ. Sensitivity analysis for contagion effects in social networks. *Sociological Methods and Research* 2011; **40**: 240-55.
14. Yang J, McAuley J, Leskovec J. Community detection in networks with node attributes. Data Mining (ICDM), 2013 IEEE 13th International Conference on; 2013: IEEE; 2013. p. 1151-6.
15. Shakya HB, Christakis NA, Fowler JH. Social network predictors of latrine ownership. *Soc Sci Med* 2015; **125**: 129-38.
16. Kim DA, Hwong AR, Stafford D, al e. Social network targeting to maximize population behaviour change: a cluster randomised controlled trial. *Lancet* 2015.
