# Supplementary material for: Exploiting social influence to magnify population-level behaviour change in maternal and child health: study protocol for a randomised controlled trial of network targeting algorithms in rural Honduras
Source: BMJ Open. 2017 Mar 10;7(3):e012996. doi: 10.1136/bmjopen-2016-012996 (PMC5353315; doi:10.1136/bmjopen-2016-012996)
Supplement: supplementary appendix [file bmjopen-2016-012996supp_appendix2.pdf]

Supplementary Appendix 2 for Exploiting Social Influence to Magnify Population-Level Behavior Change in Maternal and Child Health: Study Protocol for a Randomized Control Trial of Network Targeting Algorithms in Rural Honduras: Trellis software

Trellis

A suite of survey and social network mapping tools

Trellis is our own suite of software tools for creating, administering, and collecting survey data and mapping social networks. Trellis consists of two software applications: a web application used by administrators for survey creation and study administration, and an Android app for offline data collection.

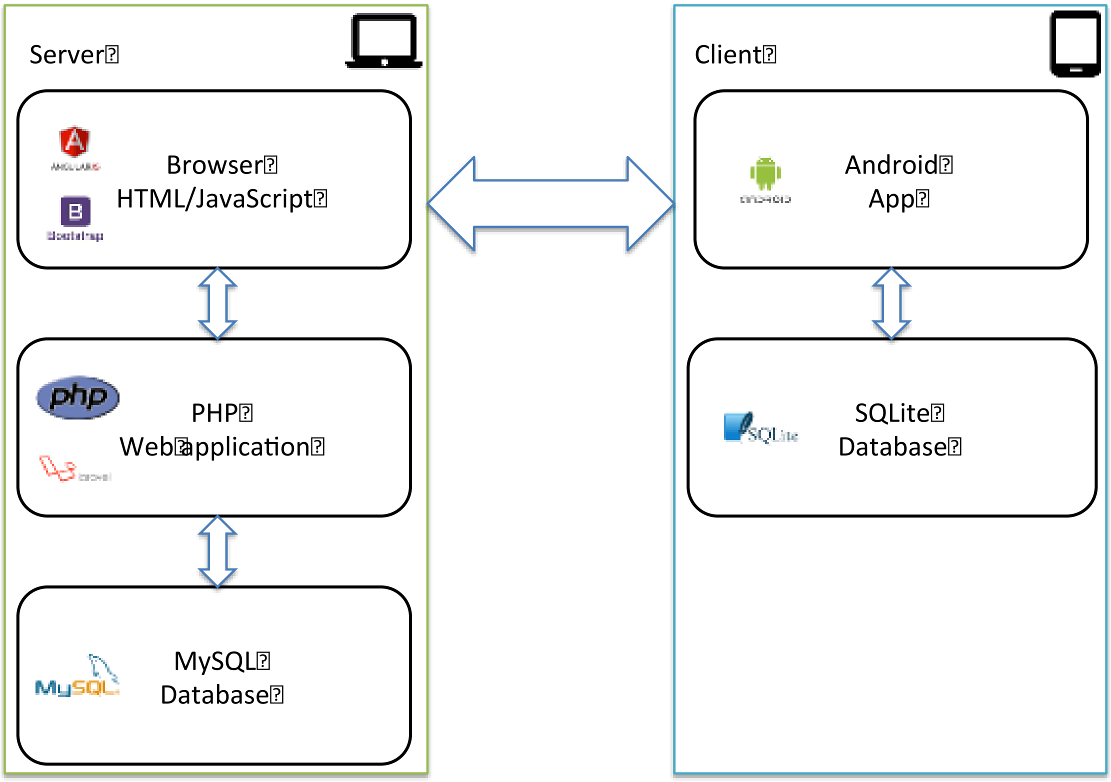

The web application is backed by a MySQL database, and written in server-side PHP, and client-side HTML and JavaScript. The client app is written in Java and runs on any Android device using the Dalvik virtual machine. Altogether, the codebase consists of over 100,000 lines of code.

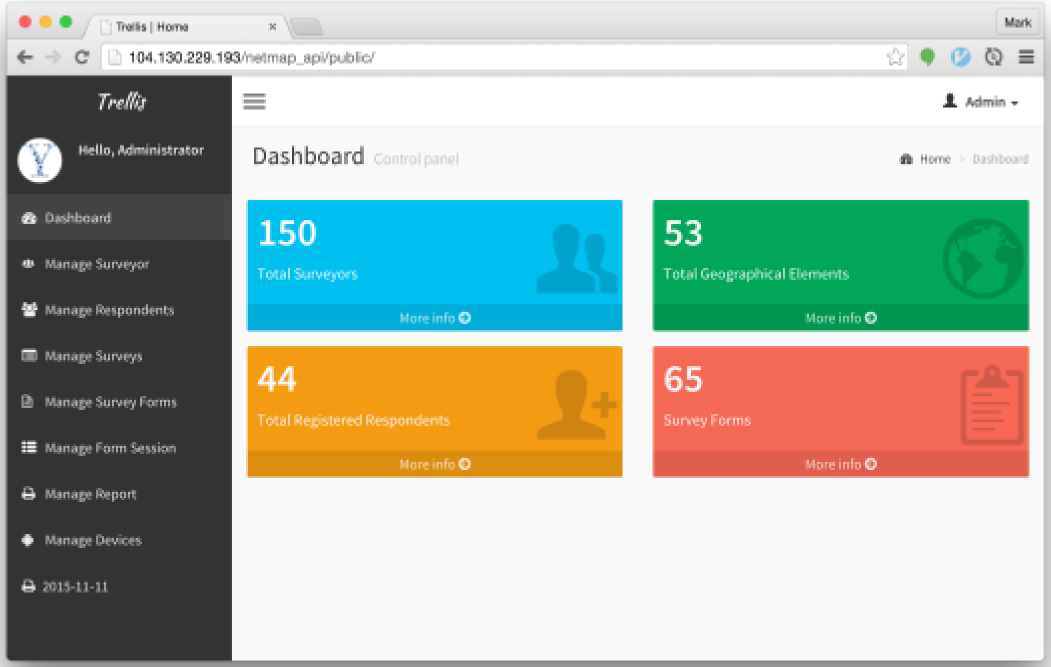

An administrator can use the web application to create new studies, register devices, add surveyors, view and edit respondents, set up study geography, add and edit survey forms, and view reports. Additionally, administrators can use satellite imagery and OpenStreetMaps to draw and tag visible buildings in order to coordinate surveyor efforts before the team is deployed to the field.

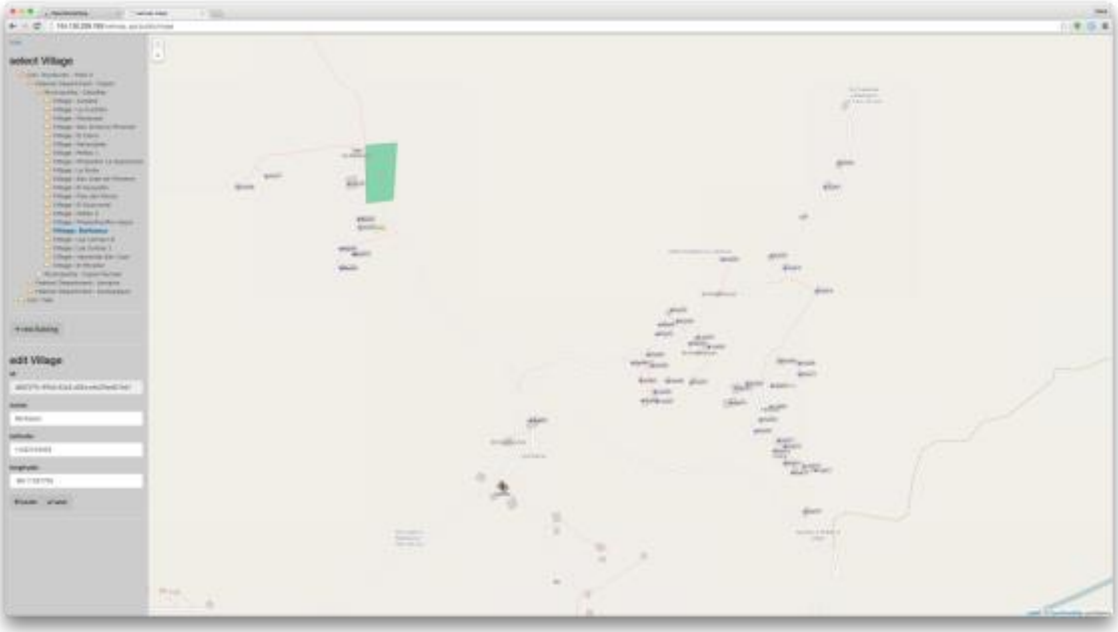

Surveyors can use the tablet app to add new buildings and households, enumerate respondents, and administer census, baseline, and follow-up surveys.

What separates Trellis from other data collection tools is its support for mapping social networks. When surveyors enumerate respondents, they capture pictures in addition to basic demographic information. After enumerating all respondents in the sample, the surveyors can ask name-generator questions such as "Who do you consider your best friends?" or "Who do you go to for health advice?" Entering a partial name in addition to known demographic information (e.g. gender, age range) will return a list of possible matches. A respondent can identify the named social contact using the pictures of returned respondents.

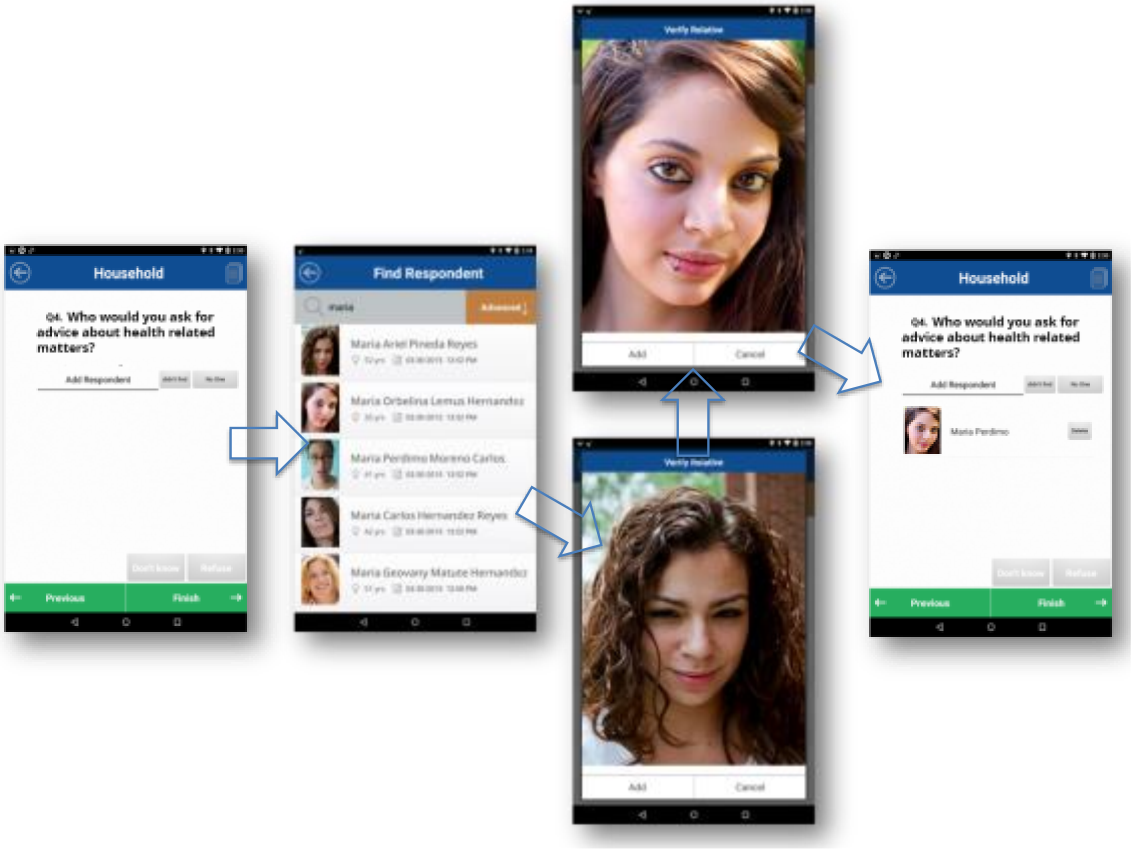

The Android app allows for offline data collection in areas without access to Wi-Fi or cellular data. At the end of the day, surveyors sync data with the server for aggregation, backup, and reporting purposes.

Surveyors in the field are currently using Trellis v1 to map the social networks of over 160 villages in Honduras. Trellis v2 is currently in development and will be released to the academic and open-source communities on completion.

Features

- A participant search feature by name, with advanced (aided) search by gender, age range, and marital status for use during network discernment data collection
- Thumbnail pictures of participants are displayed with search results
- Participant search feature includes full-screen photo for choice confirmation
- Multi-lingual user interface and survey form support
- Ability to record features of surrounding physical environment, such as photographs of buildings near the point of data collection
- Collects individual-level demographic data and assigns participants to households for synchronizing of household-level data

- Sync process allows for fully functional off-line use (i.e., multiple tablets can be used offline and data can be aggregated at a later time)
- Password-protected user login
- Participant forms page displays surveys assigned to participant along with their completion status (Unstarted, % Completed, or Finished)

Surveys in Trellis may be composed of many question types, including:

- Yes / No
- Multiple Choice (one response)
- Multiple Choice (multiple response)
- Matrix question (e.g. Likert scale)
- Text entry
- Numeric entry with range validation
- Photographic question (images taken with Android device's camera
- Relationship question (answer is one or more enrolled participants)

Trellis Admin Console features include:

- App accessible using any web browser
- Create survey forms including:
  - Adding questions
  - Translating question text into multiple languages
  - Drag and drop question reordering
  - Skip logic including multiple skips per question with skip sub-conditions
- Administer data collector's permissions:
  - Add new surveyors
  - Change surveyor username or password
  - Assign surveyors to specific geographic areas
- Add geographic locations to studies:
  - Set up a geographic hierarchy (e.g. Country / State / City / Building)
  - Add geographic elements:
    - Through a web app form
    - By clicking structures visible on satellite imagery
    - In the field (with location provide by the Android device's GPS)
