# Supplementary material for: Exploiting social influence to magnify population-level behaviour change in maternal and child health: study protocol for a randomised controlled trial of network targeting algorithms in rural Honduras
Source: BMJ Open. 2017 Mar 10;7(3):e012996. doi: 10.1136/bmjopen-2016-012996 (PMC5353315; doi:10.1136/bmjopen-2016-012996)
Supplement: supplementary appendix [file bmjopen-2016-012996supp_appendix3.pdf]

Supplementary Appendix 3 for Exploiting Social Influence to Magnify Population-Level Behavior Change in Maternal and Child Health: Study Protocol for a Randomized Control Trial of Network Targeting Algorithms in Rural Honduras: Intervention Implementation Details, Visits and Topics & Formative Research Results

Intervention implementation details:

14 visit topics have been created for the intervention. Based on the household diagnosis, the CHW selects where to start and how to advance through the program and visits can be repeated depending on reported behaviors. Topics and order are modified as the family situation changes. The materials for the CWH created for the household intervention include:

1. 4 Counseling Books
2. 3 sets of illustrated cards with information about proper diet and nutrition during pregnancy, danger signs for pregnant, birth, postpartum and postnatal periods
3. 25 stories of 3 to 5 minutes each in a 2D audio-visual format, including sound, music, and the story read aloud.

Materials are also provided to the families who partake in the intervention to reinforce key messages and motivate the practices discussed. These materials include:

1. Book of messages and agreements for the family
2. Pictures related to the messages discussed and crayons for coloring
3. House Identifier
4. Calendar with key messages for household
5. Unisex cardboard box with printed lined bearing the logo and slogan of the project.
6. Picture to hang on the wall portraying a sense of maternity lived by the whole family

The home visit is divided into four main parts 1) validation of the house; 2) introduction; 3) development; and 4) closing, and contain the following steps:

1. **Validation:** Per study requirements, the CHW records their GPS location, verifies that the people in the house match the list of people provided by YINS, and record any additional meeting attendees.
2. **Introduction:** Greeting and check in with the family to determine current situation, and introduction to the theme of the visit. As the family gathers, the CHW will play the song for the intervention, record all members present and conduct a quick survey to determine level of knowledge, attitudes and practices related to the topic. This CHW will also explain the activities to be conducted during the visit.
3. **Development:** The CHW begins by checking if the family has a priority issue they would like to discuss during the session. After discussing these issues, the CHW begins telling the "problem" story or playing the story animation on the tablet, ensuring that all family members carefully listen, look at the pictures, and answer any questions the family may have. The CHW will then tell the "positive" story, and uses the "guide" questions that accompany the story to have a dialogue about the story and negotiate behavior changes with the family. The CHW will then use complementary tools such as songs, riddles or visual aids to review main ideas with the family.
4. **Closing:** The CHW will summarize the discussion and write the agreement in the family counseling book regarding what the family wishes to practice and alternative solutions to the potential barriers identified during the visit. As a reaffirmation of the messages of the story, the last activity is to provide coloring book sheets to the family to color pictures of the key messages discussed. Additionally, the date of the next visit

will be agreed and included on the family's calendar. The CHW will play the intervention song again and thank the family again for their time before leaving.

Intervention Visits Topics

Visit Number	Household Situation	Topics Covered
Visit 0	All Households in the study	Introduction to the program, verification of household data, rapid diagnostic to determine visit plan
Visit 1	Household with woman 2/3 Months Pregnant	The importance of early prenatal care<br>Danger signs during pregnancy and active care seeking<br>Use of folic acid and prenatal vitamins
Visit 2	Household with woman 4/5 months pregnant	Planning and saving for birth (Birth Plan +: prenatal care visits, post-natal visit, birth, emergency)<br>Decisions regarding financial resources and care for sick women and children<br>Male participation during prenatal care visits
Visit 3	Household with woman 6/7 months pregnant	Importance of institutional birth<br>Danger signs during birth and active searching of medical care<br>Male participation during birth
Visit 4	Household with woman 8 months pregnant	Importance of family planning and birth spacing, informed selection of the method, participation of the male partner<br>Use of family planning methods<br>Care of the neonate<br>Male participation during birth<br>Care of the neonate: immediate breast-feeding, skin-to-skin care, dry cord care<br>Risks associated with improper cord care, use of the "fajero" and "chupon"
Visit 5	Household with woman 9 months pregnant	Care of the neonate: immediate breast-feeding, skin-to-skin care, dry cord care<br>Risks associated with improper cord care, use of the "fajero" and "chupon"<br>Post-partum care of the woman<br>Danger signs during post-partum/post-natal period and active searching of care<br>Importance of post-natal/post-partum visit within the first 3-7 days of birth<br>Male participation during birth
Visit 6	Household with neonate 0-7 days	Care of the neonate: immediate breast-feeding, skin-to-skin care, dry cord care<br>Risks associated with improper cord care, use of the "fajero" and "chupon"<br>Post-partum care of the woman<br>Danger signs during post-partum/post-natal period and active searching of care<br>Importance of post-natal/post-partum visit within the first 3-7 days of birth<br>Male participation in child care
Visit 7	Household with neonate 8-28 days	Care of the neonate: immediate breast-feeding, skin-to-skin care, dry cord care<br>Risks associated with improper cord care, use of the "fajero" and "chupon"<br>Post-partum care of the woman<br>Danger signs during post-partum/post-natal period and active searching of care<br>Importance of post-natal/post-partum visit within the first 3-7 days of birth<br>Male participation in child care
Visit 8	Household with children 1-59 months	Exclusive breast feeding for the first six months<br>Risks of "chupones"<br>Healthy baby care (danger signs, hygiene, vaccines)<br>Male participation in child care
Visit 9	Household with children 1-59 months	Knowledge of danger signs of IRAs and EDAs<br>Active searching of medical care and proper treatment<br>Proper use of ORS and Zinc<br>Male participation in child care
Visit 10	Household with WRA 10-49 years	Importance of preventing pregnancy prior to 18 years of age<br>Use of folic acid and micronutrients<br>Use of family planning methods
Visit 11	Household with WRA 10-49 years	Importance and benefits of consuming folic acid for the mom and baby prior to pregnancy<br>Importance of the pre-conception visit and male participation<br>The importance of early prenatal care<br>Reproductive life plan
Visit 12	Household with WRA 10-49 years	Importance of preventing pregnancy prior to 18 years of age<br>Ideal age for pregnancy<br>Risks associated with pregnancy prior to 18 years
Visit 13	Household with WRA 10-49 years	Self-esteem and value, life goals<br>Relationships, family communication
Visit 14	Household with WRA 10-49 years	Prevention of gender associated violence<br>Non-violent communication

Cross-cutting themes for All visits	All households	Behavior Change<br>Participation of men and the family in childcare<br>Gender and non-violent communication<br>Raising children with love and tenderness

Main Findings from Formative Research

Topic	Main Findings
**Family Planning and use of Modern Contraceptive Methods**	There are women of reproductive age that demonstrate they are in favor of family planning; however, there is evidence that work still needs to be done for complete access. For example, one factor is that some partners do not allow women to use family planning methods.<br>*"The situation makes us cry, some of the women tell us they didn't want to be pregnant"*<br>Culturally, it is believed that families should be planned after the first child, and because of this, many adolescents do not plan their pregnancies. If their partners permit it, they will use family planning methods. Of the six post-partum women interviewed, only one was more than 20 year old. The other 5 were less than 18 years old, and the newborn was their 2nd child. It is socially acceptable for adolescent women to become pregnant, but if the adolescent does not have a partner, she may not be accepted by her family and community.<br>The most commonly mentioned contraceptive methods were: the pill, injection, and IUD. The preferred method is injection, given the disadvantage of potentially forgetting to take the pill and possible perceived consequences of using an IUD.
**Folic acid (FA)**	Most participants were aware that it is important for pregnant woman to get sufficient FA, but not all the women interviewed were taking it, nor did they know the difference between FA, iron, and other vitamins. Some positive attitudes toward taking FA were identified despite the belief that it can cause spots on the skin.<br>*"If you don't take folic acid, the baby may be born with low birth weight and some type of physical deformity."*<br>*"I discontinued the treatment because it can cause my skin to spot."*
**Antenatal checkups & Warning signs during pregnancy**	Women shared two positions on antenatal care: they considered it helpful to the baby; however, if only a male doctor is available, they consider it to be shameful / embarrassing that he would see their private parts.<br>*"At first I didn't want to go, because I had never had that type of exam before, actually it was embarrassing."*<br>*Pregnant woman (247 - 250)*<br>*"In my case I decided [to go for checkup] because if you want your baby to be born healthy, you have to go."*<br>*Woman of childbearing age (210)*<br>Pregnant women generally recognize the warning signs during pregnancy and express their willingness to seek medical treatment in a health center if necessary. However, other factors, such as not have the resources to travel, may impede timely action. Given that men do not participate in prenatal visits or the negotiation of the birth plan, saving is not always in the control of the women, or possible given the economic position of the household.<br>*"Since they [men] are the ones who have the money, they are the ones who have to start saving."*<br>*"The amount saved per month appears on the paper they give you of the ID records. Supposedly it's accurate, but it's hard not to doubt: maybe I have that, but maybe there really is nothing."*
**Nutrition and rest during pregnancy**	Pregnant women eat what is available, nothing special.<br>*"Pregnant women eat what is available; usually they do not have special food to ensure that the pregnancy is healthy."*<br>An accepted social norm is that pregnant women should not rest during the day, they should only rest at night. The belief held by both men and women is that to nap (sleep) is not good during pregnancy.<br>*"Sleeping makes a woman gets sicker; she should keep working."*<br>*"Sleeping is bad, since they say it is then hard to have a baby."*
**Post-partum care**	Trends were observed in pregnant and puerperal women and grandmothers to restrict the food eaten by the woman during this period, favoring chicken broth, tortillas and cheese; and avoiding meat, vegetables and fruit in the belief that these are fresh or cold foods that could affect the woman's womb.<br>Pregnant and puerperal women and grandmothers tended to believe that during the post-partum period, the woman should protect her ears, feet, and back so they are not exposed to air and get swollen. The air conditioning in hospitals is viewed as a problem given this belief.<br>Beliefs were identified that the puerperal woman should shift her daily bath to once a week, although there were indications that they do not hold to this tendency.<br>*"You have to take special care for 42 days, after birth you bathe just once every 8 days, then 7 then 6, and then 5."*

**Newborn Care**	Good hygiene is generally maintained with newborns and it is generally recognized that when warning signs in the puerperal mother or newborn occur, it is necessary to go to the hospital or clinic.<br>Men and pregnant women reported that the practice of early attachment for breastfeeding in their personal experience is not currently happening immediately after birth. They did not show a positive or negative attitude toward the practice of early attachment for breastfeeding. Grandmothers were noted play an important role during this period. However, the use of *Chupones* is a very common practice, and introduce herbs and other liquids to the newborn.<br>*"Grandmothers take better care of the baby than the mother, because of their experience."*<br>Professional health personnel do not believe in the traditional navel care practices. In the community, the groups surveyed showed two points of view. One view accepted the health service message to leave the navel alone. And the other supported the traditional view.<br>*"These are our personal beliefs. I put it [a coin tied on with a sash] back on after it slipped off when the baby cried."*<br>*"Usually the navel is heated with something warm or covered with a sash. You have to cover and heat it so it can heal."*
**Treatment of acute respiratory illnesses (IRAs) and acute diarrheal infections (EDAs)**	Treatment of IRAs includes the use of teas with liquorish, avocado, eucalyptus, chamomile, guava buds cure the IRA. Many times, with the first symptom of cough, children are self-medicated with antibiotics.<br>Families have knowledge of how to prevent diarrhea, however, they do not describe specific danger signs associated with dehydration. Herbal teas to treat diarrhea are used in children and infants. Children as also self-medicated with anti-biotics.<br>Families know about the use of ORS and will go to the health center if diarrhea is persistent.<br>Families do not know the use of zinc as a treatment for diarrhea, this implies that they do not request it in the health center and that do not comply with treatment when they have received the drug.
**Involvement of men**	Pregnant women, women of childbearing age and grandmothers indicated that men (during their wife's pregnancy), usually: Go with the pregnant woman to the doctor but do not accompany her in to the appointment; Buy the medication that the pregnant woman needs; and Show affection to the pregnant woman and baby.<br>The men expressed willingness to do tasks / chores that the pregnant woman is unable to do, however women said culture might inhibited this practice.<br>*"I even carry the water because it is too heavy for her; we have to bring it from the well, there is no water nearby.*<br>*"Some men are chauvinists, some are embarrassed [to accompany women to health center] because they are not used to doing it, culture perhaps."*
**Relationship with Local Health Providers**	The primary need identified communication is to improve the quality of the relationship between MANCORSARIC and Maya-Chorti communities and crossbred to build a real synergy between both parties to facilitate a gradual improvement in neonatal outcomes<br>Although there is general widespread belief in institutional births, some people question whether it should be obligatory, disrespecting the opinion and desire of each individual.
